# Genome wide identification, characterization of upstream protein kinases involving CBF dependent pathway and antioxidant enzyme analysis in Watermelon (*Citrullus lanatus*) under brassinosteroid and cold treatment

**DOI:** 10.1007/s12298-026-01824-1

**Published:** 2026-10-12

**Authors:** Ijaz Yaseen, Hoy-Taek Kim

**Affiliations:** Department of Horticulture, Sunchon National University, 255, Jungang-ro, Sunchon-si, Jeollanam-do 57922 Republic of Korea

**Keywords:** Tolerance, Sensitive lines, Superoxide dismutase, Peroxidase, Electrolyte leakage

## Abstract

Brassinosteroids (BRs) are steroidal plant hormones involved in regulation of physiological and molecular processes to ameliorate various biotic and abiotic stresses. Exogenous application of BRs to improve stress tolerance in plants has recently become a high research priority. The involvement of these steroidal hormones in upregulation of stress-related defense genes and their cross talk with other metabolic pathways like CBF dependent and independent pathways are gaining importance. In this study, the primary and master regulator protein kinases (PKs) e.g. BRASSINOSTEROID INSENSITIVE1 (BRI) and BR SIGNALING KINASES (BSK) were analysed. Initially, ClaBRI-ClaBSK gene families were identified and characterized after RT-PCR analysis under cold treatment (CT). These findings were subsequently confirmed in the cold-sensitive lines (PI526233 and SW54) under BRs and cold simultaneous treatment (BRs + CT). Additionally, bio-physiochemical properties, phylogenetic analysis, gene structure, cis element identification and chromosome localization were also analyzed. The activities of superoxide dismutase (SOD), peroxidase (POD) was significantly increased in tolerant and sensitive lines under both treatments (CT and BRs + CT) as compared to sensitive lines under only CT treatment while the electrolyte leakage (EL) was observed to be opposite to these enzymes’ activities for same treatments. In RT-PCR results, the genes *ClaBRI2*,* ClaBSK1*,* ClaBSK4* and *ClaBSK8* showed positive expressions in case of same treatments mentioned above. Furthermore, the upregulation of *ClaHDA6* (positive regulator of cold tolerance) and the downregulation of *ClaBIN2* (negative regulator of cold tolerance) in earlier times impart the significance of the present study. These results suggest that BRs application may contribute to improved cold stress tolerance in watermelon.

**Supplementary Information:**

The online version contains supplementary material available at https://doi.org/10.1007/s12298-026-01824-1.

## Introduction

Climate change is creating severe pressure on world farming by increasing the frequency, length, and overlap of abiotic stresses such as flooding, salinity, drought, temperature extremes, high CO_2_, organic pollution, metal toxicity and nutritional imbalance etc. (Raza et al. 2026a, b; Silverwood et al. 2025). However, the growth, development, yield, and geographic dispersion of plants are all greatly impacted by low-temperature stress (Adhikari et al. 2022; Guan et al. 2023). Low temperature may trigger oxidative damage, enhance membrane permeability, hinder photosynthesis, and even result in widespread cell death, according to mounting data (Chen et al. 2025; Raza et al. 2024). Watermelon (*Citrullus lanatus*) is an extremely nutritious fruit consists of 93% water with low concentrations of protein, fat, minerals, and vitamins. Its enormous consumption worldwide can be attributed to its widespread recognition as a functional food (Assefa et al. 2020). Cucumber, watermelon, melon, and squash are the four primary cucurbit crops. Of these key crops, watermelon has the largest worldwide consumption (Wehner et al. 2020). Watermelon needs a warm growing season of 75 to 95 days from planting to harvesting, and they are primarily grown in tropical and subtropical areas. Watermelons can withstand temperatures as low as 18 °C and as high as 32 °C, however their ideal growth temperature is between 21 and 29 °C (Noh et al. 2013; Shirani Bidabadi and Mehralian 2020). Low temperature has a detrimental effect on the reproductive and vegetative stages of the watermelon plant life cycle. During reproductive development, low temperature stress can delay blooming and promote floral abscission, pollen sterility, pollen tube shortening and deformation, and reduced fruit set, which affects yield (Nishiyama 1970; Waraich et al. 2012). Low temperature can reduce stomatal conductance and leaf water content during the vegetative development stage, resulting in smaller leaves and shoots (Rodríguez et al. 2015).

Literature studied that Cold receptors on plant cell membranes typically detect low temperatures and cause calcium influx, which in turn activates the calcium (Ca^2+^) signaling cascade network (Ma et al. 2015) and the protein kinase-regulated phosphorylation cascade (Zhao et al. 2017). This, in turn, activates the downstream ICE1-CBF-COR signaling cascade pathway, which is also called CBF dependent pathway (Gusain et al. 2023). Plants exposed to one kind of stress have shown the ability to affect how they react to other stressors, suggesting the existence of intricate stress response networks that have been shaped by their long-term coexistence with a variety of environmental dynamics. Transcription factors show up in these networks as crucial regulators of genes that respond to stress, making them attractive options for boosting crop resilience (Liu et al. 2024). The coordination of stress responses by transcription factors is a dynamic process involving multiple gene families (Wang et al. 2026).

The most significant and typical plant response to cold stress is thought to be the ICE1-CBF COR pathway. Transcription factors orchestrate cold stress responses by integrating upstream signals and activating defense gene expression (Liu et al. 2019). Nonetheless, the ICE1-CBF-COR cascade’s transcriptional activation is a complex network that includes the direct or indirect control of transcription factors (TFs) (Zhang et al. 2022a; Kamble 2024), post-translational modifications (PTMs) (Ding et al. 2019), light stimuli (Jiang et al. 2020; Zhang et al. 2022b), circadian rhythm factors (Kidokoro et al. 2021, 2023), and interacting proteins (Jin et al. 2017; Lee et al. 2021).

Organisms must integrate signals in order to regulate their growth, development, and stress reactions. As endogenous signals, phytohormones are crucial in mediating a number of processes in reaction to external stimuli (Yoshida et al. 2025). BRs, a significant class of polyhydroxylated sterols present in plants, are extensively employed in agriculture to boost yield and improve plant resilience to environmental stresses (Clouse 2011). BRs control the expression of genes on several signaling pathways in response to cold stress. BR-regulated transitory cold stress and cold stress acclimatization are exemplified by the ICE-CBF-COR route and the non-CBF dependent pathway, respectively. However, the main and master regulator (BRI1 and BSK1) of cold stress signaling cascades trigger by BRs are analyzed, linked with BIN2 and HDA6 proteins in this study.

The cell surface receptor kinase (BRI1)’s extracellular structural domain detects BRs and sets off a signal transduction cascade to control the transcription of downstream genes (Kim and Wang 2010; Wang 2012; Kim et al. 2009). The co-receptor kinase BRI1-ASSOCIATED KINASE1 (BAK1) is recruited during BRI1 activation, and when the extracellular domains of BRI1 and BAK1 bind, BRI1 kinase inhibitor 1 (BKI1) separates from the cytoplasm membrane, which subsequently causes the kinase domains of BRI1 and BAK1 to be mutually phosphorylated, activating them (Tang et al. 2008).

After activation BRI1 phosphorylates the CONSTITUTIVE DIFFERENTIAL GROWTH1 (CDG1) and BSKs families to activate them (Kim et al. 2011; Sreeramulu et al. 2013; Wang et al. 2002). The PP1-type phosphatase BRI1-SUPPRESSOR1 (BSU1) is then activated by BSKs and CDG1, which dephosphorylates and inhibits BRASSINOSTEROID INSENSITIVE2 (BIN2) (Sreeramulu et al. 2013; Yin et al. 2002). The phosphorylation inhibition of BIN2 on downstream transcription factors BRASSINAZOLE-RESISTANT1 (BZR1) and BRI1-EMS-SUPPRESSOR1 (BES1) is alleviated when activated BSU1 dephosphorylates the inactivating negative modulator BIN2 (Yin et al. 2002; Chen et al. 2023).

By regulating photosynthesis, increasing antioxidant capacity, and encouraging osmotic adjustment in the face of drought, salinity, and low temperature stress, BRs have been demonstrated in studies to improve plant stress tolerance (Wang et al. 2010a, b; Zhang et al. 2022c; Mu et al. 2022). Exogenous BRs can improve photosynthesis, cold and salt tolerance by scavenging reactive oxygen species and reducing oxidative damage in rice seedlings (Yang et al. 2025). The growth inhibition of seedlings of rice under cold water stress was considerably reduced by exogenous brassinolide (BL), which also increased the activities of antioxidant enzymes like SOD and POD and efficiently increased germination rates and biomass (Yang et al. 2025). It’s interesting to note that no research has been done on how exogenous BR application affects watermelon germination, seedling biochemical traits, or how BRs control CBF-dependent and independent pathways during cold treatment.

Therefore, this study was conducted to identify the BRs regulated downstream signaling cascades, including primary PKs, particularly BRI and BSK and biochemical factors which offers a novel approach to enhancing watermelon cold resistance and serves as a theoretical basis for the subsequent identification of important genes in the watermelon cold resistance mechanism. In this study, two *ClaBRI* and eight *ClaBSK* genes were identified in watermelon and the comprehensive analyses including the gene structures, conserved motifs, cis elements identification and phylogenetic analysis were performed. The expression patterns of these BRs regulated PKs, *ClaBRIs*, *ClaBSKs*, *ClaBIN2* and *ClaHDA6* were also analyzed when CT and BRs + CT were applied to both tolerant and sensitive lines. In addition, the activities of antioxidant enzymes e.g. SOD and POD and electrolyte leakage were also observed in the presence of the above given treatments. This work might be revealed the molecular activities and characterizations of these PKs and targeted genes for the cucurbit breeding program.

## Materials and methods

### Plant materials, cold treatment and expression data

In this experiment, the “SW66 and PI254744” (tolerant lines) and “SW54 and PI526233” (sensitive lines) were used as the experimental material. The first pair of watermelon lines (SW66 and PI526233) was discussed as group 1 and second pair (PI254744 and SW54) was categorized as group 2. These tolerant and sensitive lines were selected on the basis of preliminary screening and physiological analysis as described in previous research (Yaseen et al. 2025). These watermelon lines were sown 1^st^ in seedling starter tray in growth chamber (28 °C, 16 h light/8 h dark, 70–80% humidity) and transferred to 10 cm pots at three leaf stage. At four leaf stage, the watermelon sensitive lines “SW54 and PI526233” under CT (10 °C) served as treatment one while the same lines under BRs + CT was considered as 2nd treatment. The brassinosteroid was applied in the following concentrations: 0.01 mg L^−1^, 0.1 mg L^−1^ and 1 mg L^−1^ by spraying method as preliminary study. However, 0.1 mg L^−1^ concentration was found suitable and selected for further analysis. The watermelon tolerant lines “SW66 and PI254744” were categorized as control under cold treatment. After the phenotypic analysis, leaf samples were collected from different plants of all treatments under the following time periods: 0 h, 6 h, 12 h, 24 h, 3d and 6d, frozen in liquid nitrogen and stored at -80 °C. Moreover, independent biological replicates were collected destructively at each time point which means the total 18 biological replicates (three different samples for each time point) and three experimental treatments were performed respectively.

Using the manufacturer’s First-Strand cDNA Synthesis Kit, 1 µg of RNA was reverse transcribed to produce the first strand of cDNA after total RNA was isolated from the sample using Trizol reagent (Takara, Japan). Primer Premier 3 software (v3.00) was used to create the primers for the *ClaBRI* and *ClaBSK* genes, and qRT-PCR was used to assess expression levels (Table S1). The reaction mix contains 5 µL AceQ qPCR SYBR Green Master Mix, 0.5 µL upstream primers, 0.5 µL downstream primers, 1 µL cDNA, and 3 µL with ddH2O. The qRT-PCR procedure was set up as follows: stage 1 involved 30 s of initial denaturation at 95 °C; stage 2 involved a circular reaction at 95 °C for 10 s and 60 °C for 30 s, 40 cycles; and stage 3 involved a melting curve at 95 °C for 15 s and 60 °C for one minute. Each sample’s average threshold cycle (Ct) was computed, the 2^−∆∆CT^ technique was utilized to assess the transcript abundance of genes (Livak and Schmittgen 2001) and β-actin was employed as an internal control.

### Identification of *BRI*-*BSK* gene family members in cucurbitaceae

We downloaded the BRI-BSK proteins sequences of *Arabidopsis thaliana* from NCBI database (https://www.ncbi.nim.nih.gov, accessed on August 12, 2025). Genome-wide identification approaches, including sequence homology-based methods, are fundamental for characterizing gene families involved in stress responses (Wang et al. 2025). These BRI-BSK proteins of *A. thaliana* were BLAST searched (protein-protein) against cucurbits blast database available on cucurbit genomic database (http://cucurbitgenomics.org/, accessed on August 12, 2025). The candidate BRI-BSK proteins were obtained from (*Cucumis sativus*, diploid, 2*n* = 14, *Cucumis melo*, diploid, 2*n* = 24, *Momordica charantia*, diploid, 2*n* = 22, *Citrullus lanatus*, diploid, 2*n* = 22, *Cucurbita moschata*, allotetraploid, 2*n* = 40) (Tables S2 and S3). ExPASy (https://web.expasy.org/protparam, accessed on October 15, 2025) was used to calculate the biochemical parameters, including the molecular weight (Mw), isoelectric point (pI), amount of amino acids, instability index, aliphatic index, and Grand average of hydropathicity (GRAVY).

### Chromosomal localization analysis

The Cucurbit Genomic Database (http://cucurbitgenomics.org/, accessed on August 12, 2025) provided the locus positions of all *BRI-BSK* genes as well as the beginning and ending points of their chromosomes. The software GGT2 (Graphical Genotypes) is used to display the locations of genes on chromosomes.

### Evolutionary analysis

Using the Program ClustalW in the software MEGA11, all protein sequences of cucurbitaceae BRI-BSK were compared, and the phylogenetic tree of the cucurbitaceae BRI-BSK family was constructed using the neighbor-joining (NJ) method and the self-help method of phylogenetic experiments (Bootstrap method, Bootstrap = 1000, and the *p*-distance model). The tree was visualized and optimized through the iTOL (https://itol.embl.de/, accessed on August 12, 2025).

### Gene structure, and conserved motif

The MEME website (https://meme-suite.org/meme/tools/meme, accessed on August 12, 2025) was used to evaluate and identify the conserved motifs of BSK amino acids after gff3 file of watermelon was downloaded from the Cucurbitaceae database in order to study the genetic structure of *BRI-BSK* genes.

The conserved domains were collected from Conserved Domain Database (CDD) published on (https://www.ncbi.nim.nih.gov/Structure/cdd/cdd.shtml) and gene structure evaluated by Tbtools software.

### Identification of *cis*-elements on *ClaBRI* and *ClaBSK* promoter in watermelon

The watermelon genome database (97103_genome_v2.5.fa.gz: http://cucurbitgenomics.org/) provided the complete watermelon genome data. TBtools was used to extract the 2.3 kb sequences upstream of the transcription start site of *ClaBRI* and *ClaBSK*. The PlantCARE website (http://bioinformatics.psb.ugent.be/webtools/plantcare/html/) was used to analyze the cis-elements in the promoter regions of *ClaBRI* and *ClaBSK* genes (Li et al. 2020).

### Antioxidant enzyme activities and electrolyte leakage test

Antioxidant enzyme activities (SOD and POD) were determined according to the method described by (Wang et al. 2010a, b) in units per gram fresh weight. The activity of SOD was measured by its capacity to inhibit the photochemical reduction of nitroblue tetrazolium (NBT). In contrast, POD activity was determined by monitoring the rate of color change as the enzyme catalyzes the oxidation of a substrate like guaiacol in the presence of hydrogen peroxide (H₂O₂). Electrolyte leakage assays were performed as described by (Li et al. 2017). After the cold stress experiment, seedlings were placed into 45 mL tubes containing 30 mL of deionized water, whose electrical conductivity (EC) was S0, and shaken for 15 min at 22℃ before their EC were measured, giving S1. After detecting S1, the samples were boiled for at least 15 min and shaken at 22℃ for another 20 min before their EC were measured again, giving S2. The electrolyte leakage was calculated as:

$$\left( {{{\rm{S}}_1} - {{\rm{S}}_0}} \right)/\left( {{{\rm{S}}_2} - {{\rm{S}}_0}} \right).$$

### Statistical analysis

Three-way analysis of variance (ANOVA) was used to statistically assess the means of the data from three RT-qPCR biological replications. The statistical software IBM SPSS statistics 25 was utilized to evaluate mean differences at the 0.05 probability level using Tukey’s Honestly Significant Difference (HSD) post hoc test.

## Results

### Physiological observation

**Fig. 1:**
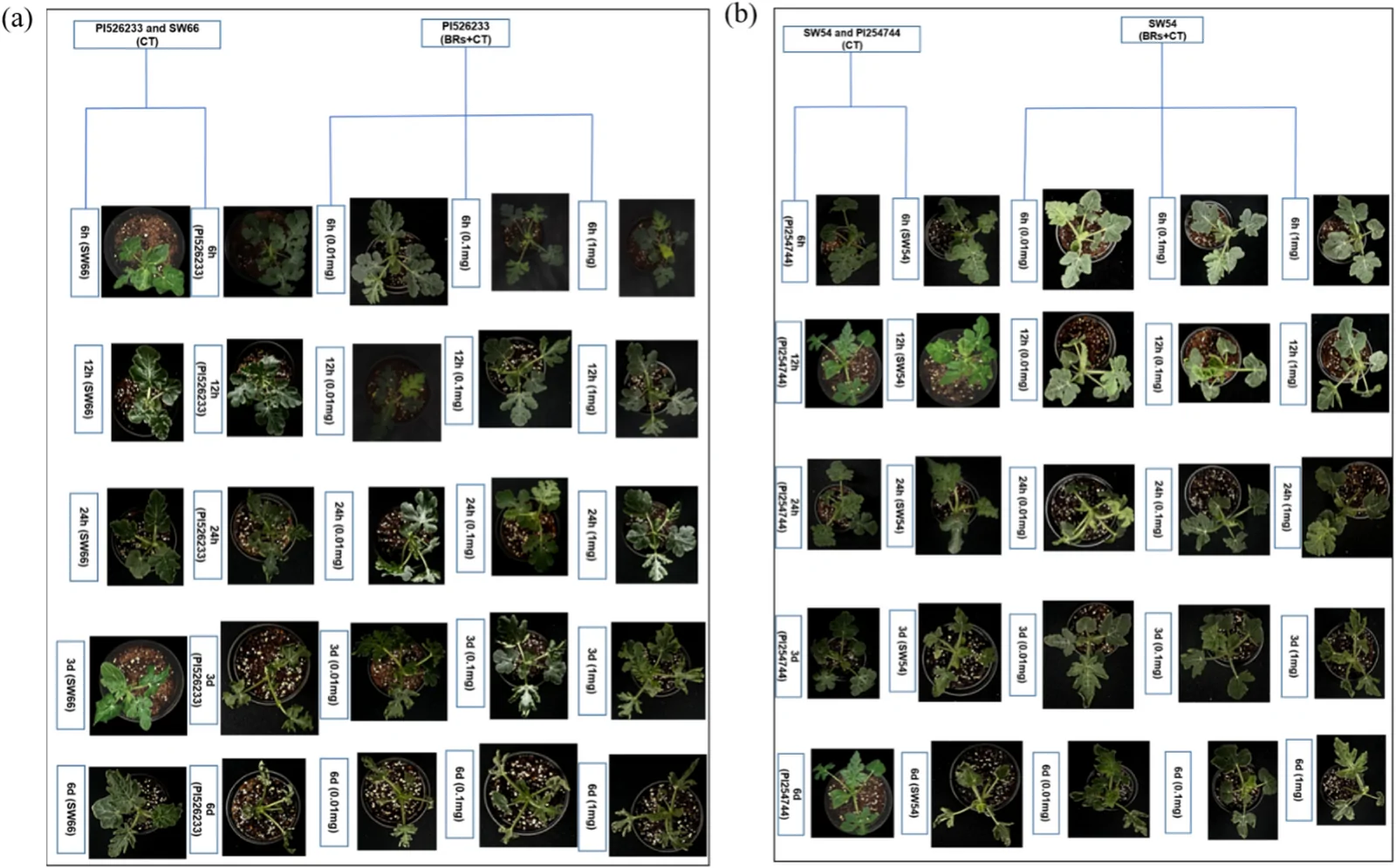
CT and BRs + CT treated tolerant and sensitive watermelon lines represented as group 1 (SW66 and PI526233) (**a**) and group 2 (PI254744 and SW54) (**b**). According to the time points e.g. 6 h, 12 h, 24 h, 3d and 6d, the six biological replications were used. Brassinosteroid were sprayed to watermelon at 4 leaf stage according to following concentrations 0.01 mg L^−1^, 0.1 mg L^−1^ and 1 mg L^−1^

The watermelon sensitive lines showed better response (less wilting and dying of lower leaves) under 0.1 mg.L^−1^ concentration as compared to other concentrations e.g. 0.01 mg.L^−1^ and 1 mg.L^−1^, showed severe wilting of upper leaves and ultimate dying of lower leaves (Fig. 1). Hence, 0.1 mg L^−1^ was found to be suitable for effective/positive response for sensitive lines against cold stress in this study. It was found that low temperatures significantly reduced the growth rate (leaf and stem size) and showed curling, wilting and dying in the later stages of watermelon sensitive lines (PI526233 and SW54) under CT. However, the opposite growth rate was observed in the tolerant lines (PI254744 and SW66) under CT except some leaves showed curling in later stages. Under BRs + CT treatment to sensitive lines, the normal growth rate and healthy leaves condition were observed at all-time points like in cold tolerant lines.

### Identification and chromosomal localization of *BRI*-*BSK* gene family members in watermelon

**Fig. 2:**
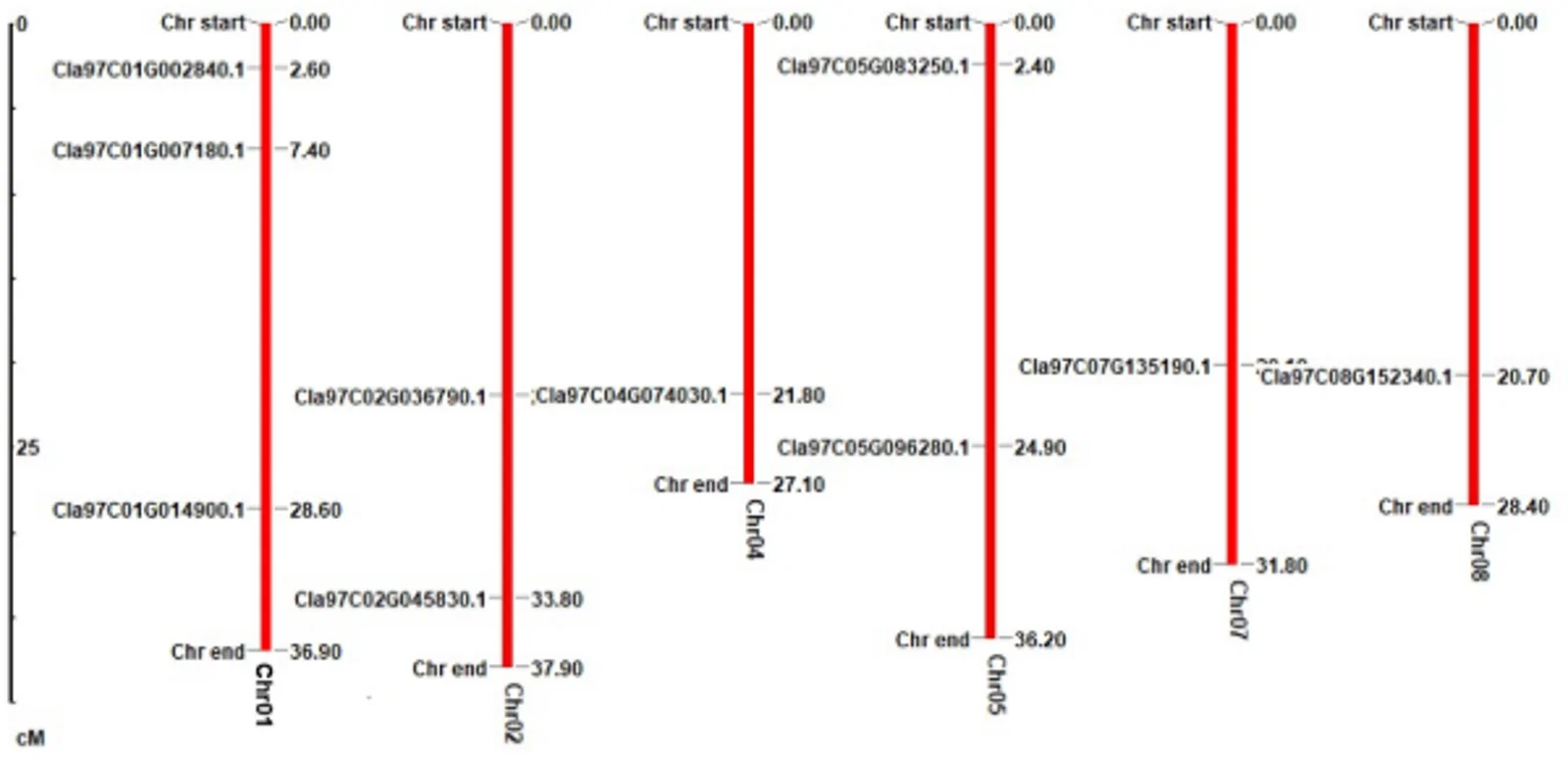
Localization of *ClaBRI*-*ClaBSK* genes on the watermelon chromosomes. The chromosome number was labelled at the bottom of each chromosome. The left scale represents the length of chromosomes, and scale is expressed in centimorgan (cM)

In this study, a total of 2 *ClaBRI* and 8 *ClaBSK* proteins were identified in watermelon. Table 1 shows the individual names of the corresponding ClaBRI and ClaBSK proteins, number of amino acids, theoretical isoelectric point (pI), and molecular weight, instability index, aliphatic index and GRAVY (Grand Average of Hydrophilicity). The length of proteins was in ranges, from 486 to 1199 amino acids, and their molecular weights (Mw) ranged from 54.422 to 129.97 kDa. The isoelectric point (pI) values of proteins ranged from 5.52 to 7.5. The instability index and aliphatic index values were found to be 39.16 to 43.86 and 75.01 to 110.45. Notably, all identified ClaBSK proteins have GRAVY values less than 0, which means these proteins are hydrophilic. All ClaBRI and ClaBSK proteins were predicted to be located in the nucleus. The localization of *ClaBRI* and *ClaBSK* genes on the chromosomes in watermelon was mapped using GGT2 software. As shown in Fig. 2, the *ClaBRIs* of watermelon were localized only on chromosome 1 whereas the *ClaBSKs* were localized on 6 chromosomes, of which there were 2 *ClaBSKs* on each of chromosomes 02 and 05. The rest 4 ClaBSKs were distributed separately on chromosomes 01, 04, 07 and 08.

**Table 1:** Protein parameters of *ClaBRI*-*ClaBSK* gene families

Accession numbers	Number of amino acids	Theoretical pI	Molecular weight (kDa)	Instability index	Aliphatic index	Grand average of hydropathicity (GRAVY)
ClaBRI1	940	5.69	103.12	42.93	110.45	0.064
ClaBRI2	1199	5.93	129.97	30.49	95.39	− 0.034
ClaBSK2	542	5.52	60.85	43.86	75.28	− 0.352
ClaBSK8	509	6.20	57.04	39.16	75.01	− 0.446
ClaBSK6	506	5.35	56.68	43.03	81.98	− 0.384
ClaBSK4	585	7.51	65.93	39.9	75.76	− 0.341
ClaBSK3	542	5.62	61.08	42.53	79.78	− 0.305
ClaBSK5	491	5.54	55.27	40.86	85.01	− 0.341
ClaBSK1	502	5.84	56.54	39.88	84.56	− 0.306
ClaBSK7	486	5.75	54.42	42.97	82.57	− 0.355

### Evolutionary analysis of *BRI-BSK* gene families in cucurbitaceae

**Fig. 3:**
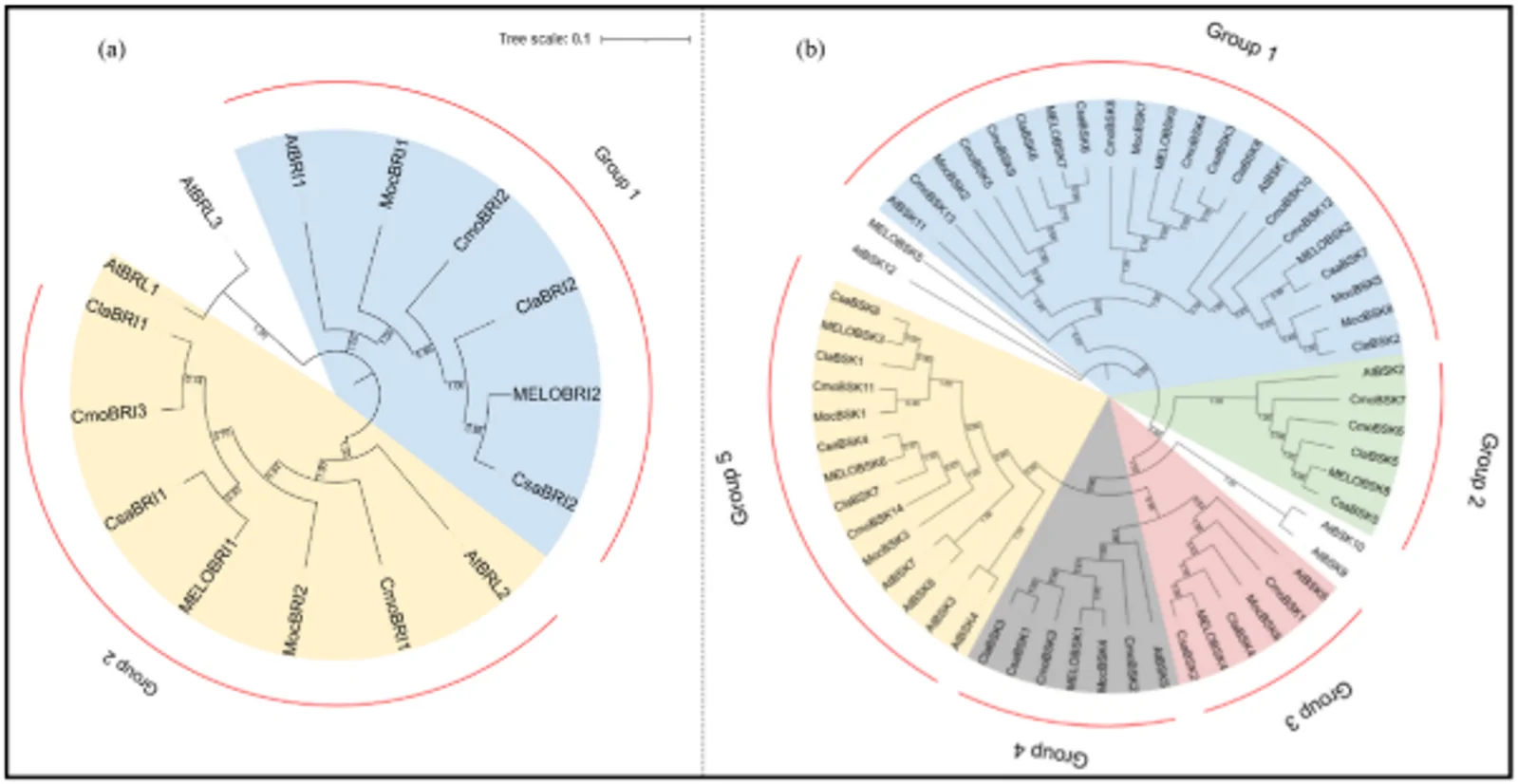
Phylogenetic relationships of BRI (**a**) and BSK (**b**) proteins from cucumber, melon, bitter gourd, watermelon, winter squash (pumpkin) and Arabidopsis. All BRI and BSK proteins in the six species were clustered into two and five groups represented by red circular lines for Groups 1 to 5, respectively. The generated phylogenetic tree included 4 BRI and 12 BSK proteins from Arabidopsis, 2 and 9 from melon, 2 and 8 from watermelon, cucumber and bitter gourd, 3 and 14 BRI-BSK from pumpkin

To better explore the evolutionary relationship of BRI-BSK proteins, a phylogenetic tree was constructed using MEGA12 (ClustalW, NJ methods) for a total of 15 BRI and 59 BSK members as study objects, of which 2 BRI and 8 BSK proteins belonged to Watermelon and rest of them in other cucurbits and *A. thaliana* described in Fig. 3. These proteins were divided into two and five distinct groups (BRIs and BSKs) according to the bootstrap values and phylogenetic topology: Group I consisted of 6 BRI members and Group II consisted of 7 BRI members in the case of BRI phylogenetic analysis. Likewise, Group I consisted of 22 BSK members, Group II consisted of 6 BSK members, Group III and Ⅳ consisted of 7 BSK members and Group Ⅴ consisted of 14 BSK members for BSK phylogeny. Each group contain ClaBRI and AtBRI, ClaBSK and AtBSK members but have less distant relationship among them. This finding implies that the BRI-BSK family expanded following dicot divergence. The close evolutionary relationship could be found between melon with cucumber and watermelon with pumpkin for BRI evolutionary analysis. However, the close evolutionary relationship could be found between cucumber with melon and watermelon for BSK phylogeny. Likewise, the same relationship was found between watermelon with bitter gourd, bitter gourd with melon, Arabidopsis with Arabidopsis and pumpkin with bitter gourd in BSK genes. However, AtBRL1 and AtBRL3, AtBSK9 and AtBSK10 belonged to the outliers/outgroups. Likewise, CmBSK5 and AtBSK12 were observed to be separate outliers.

### Gene structures and conserved motifs

**Fig. 4:**
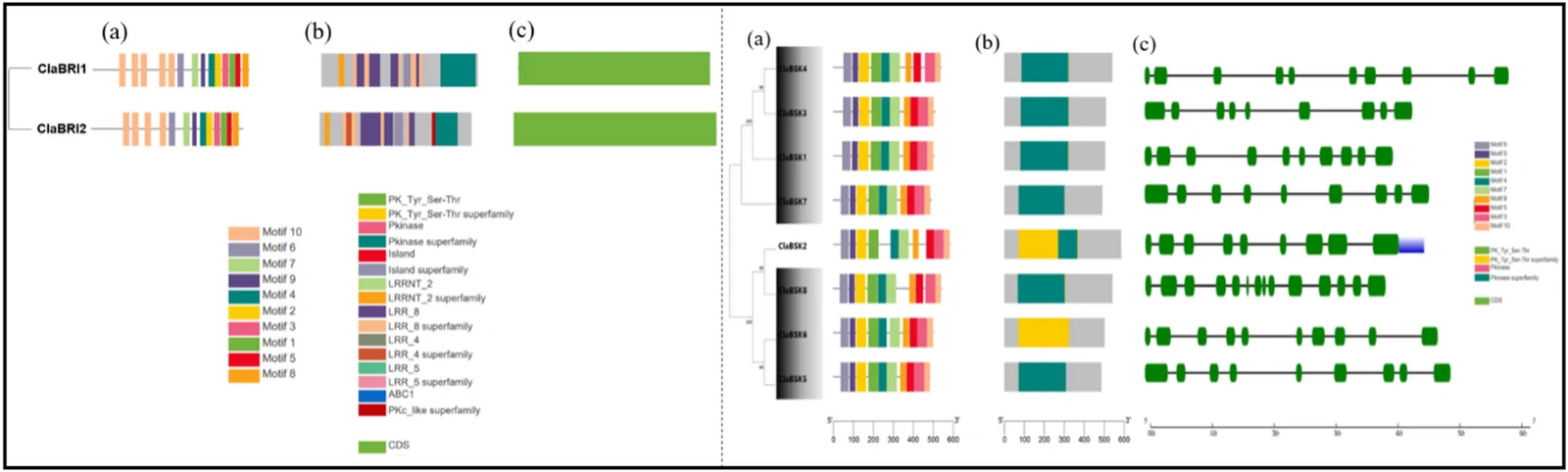
Conserved motifs distribution (**a**) Conserved domains (**b**) Gene structures (**c**) of BRI and BSK proteins in watermelon. The CDS were represented by green boxes, and the introns were represented by black lines. Motif analysis was conducted using the MEME software and TBtools software (v1.108). Different color boxes represented various types of conserved motifs

The structural characteristics of *BRI-BSK* genes, consisting of conserved motifs, conserved domains, CDS (coding DNA sequences), UTRs (untranslated regions), and introns, were described in the Fig. 4. The conserved motifs of all BRI-BSK amino acids only in watermelon were analyzed using the MEME website. It was found that the number of motifs in all BRI-BSKs were found maximum (10) (Fig. 4a). Out of the all observed domains e.g. PK-Tyr-Ser-Thr, PK-Tyre-Ser-Thr superfamily, Pkinase, PKinase superfamily, LRR (2,4,5 and 8) etc., PKinase superfamily and LRR8 showed dominant character and probably have the most vital roles in the molecular function (Fig. 4b). As shown in Fig. 4c, BRI members consisted of only 1 exon region while BSK family members contained at least 9 exons and up to 14 exons, with at least 8 introns and up to 13 introns. These findings suggest that these motifs were highly conserved and might play the important role in the BRI-BSK gene family.

### *Cis*-elements identification of *ClaBRI and ClaBSK* gene promoters in watermelon

According to Verma et al. (2016), cis elements are crucial for controlling gene expression in response to biotic and abiotic stressors. ABA-responsive elements (ABRE), Myeloblastosis (MYB), MYB-Binding site (MBS), defense-related TC-rich repeats, Salicylic acid (SA)-responsive Tricarboxylic acid (TCA) elements, low-temperature-responsive LTR, ethylene-responsive element (ERE), and jasmonic acid (JA)-responsive CGTCA-motif are among the cis-regulatory elements in *ClaBRI* and *ClaBSK* promoter regions that are associated with stress and hormone responses (Fig. S1). The ethylene-responsive cis elements, or EREs, typically interact with the dehydration-responsive elements, or DREs, to protect the plant from abiotic stressors like cold temperatures.

### Expression profiling of brassinosteroid regulated genes (BRGs) under CT and CT + BRs

To investigate the comparison under the given treatments discussed in methodology section, qRT-PCR was used to detect the expression levels of BRGs.

### RT-PCR for 1st group parent lines (SW66 and PI526233)

**Fig. 5:**
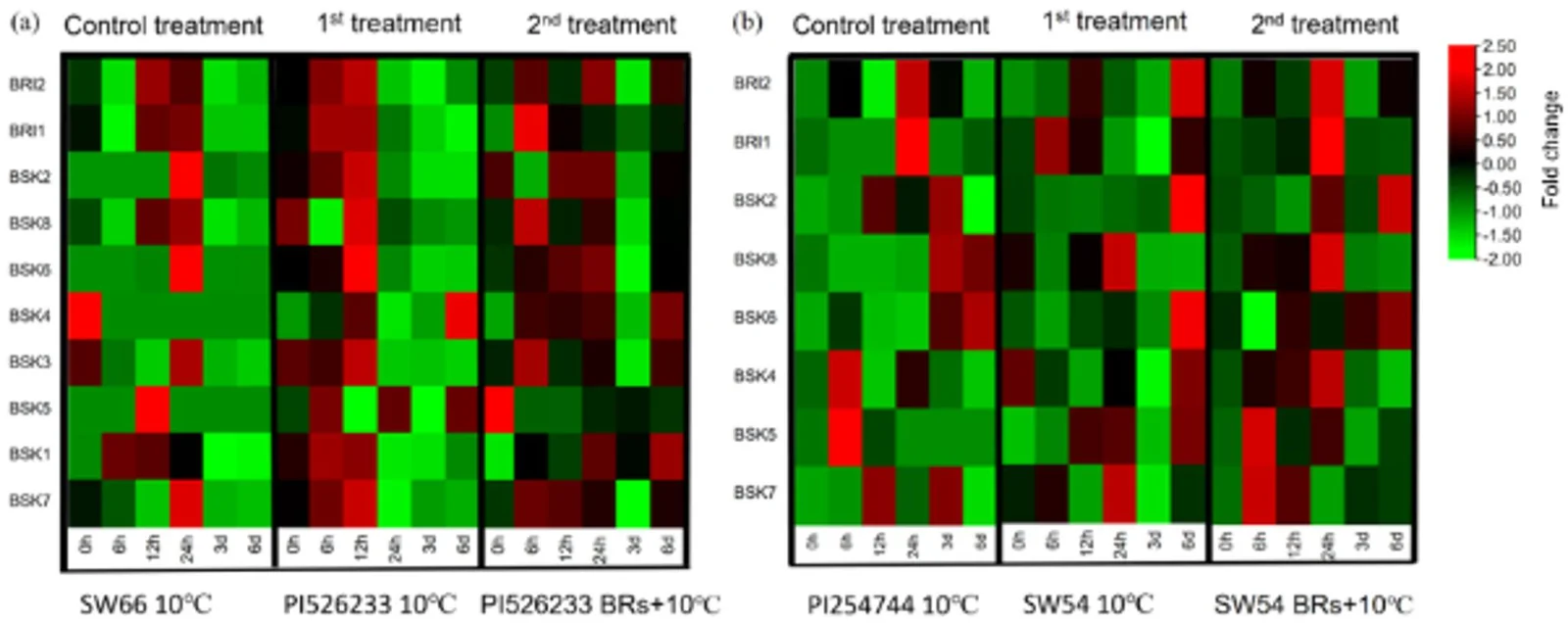
Heatmap showing the expression patterns of *BSK* genes in watermelon leaves of Group 1 (SW66 and PI526233) (**a**) and Group 2 (PI254744 and SW54) (**b**) at different time points (0h, 6h, 12h, 24h, 3d, and 6d). Gene expression levels are presented as log_2_ fold-change values relative to the 0 h control. Green indicates downregulated or lower expression, whereas red indicates upregulated or higher expression

If we compare the 1st treatment (CT) with 2nd treatment (CT + BRs), the given *BSK* genes showed some expression at 12 h and 24 h in the case of 1st treatment while the same genes showed higher expression at almost all time points after 2nd treatment. (Fig. 5a). However, the *BSK* genes also showed higher expression at 6 h, and 24 h in the case of control treatment (the tolerant line, SW66). The genes *ClaBRI1, ClaBRI2, ClaBSK1, ClaBSK4, ClaBSK6* and *ClaBSK8* were expressed significantly positive at all-time points for 2nd treatment as compared to 1st treatment. However, the *ClaBRI2, ClaBSK1* and *ClaBSK8* showed positive expression at initial time points, 12 h and 24 h for control treatment.

### RT-PCR for 2nd group parent lines (PI254744 and SW54)

Likewise, the higher expression patterns of BSKs were found at 6 h and 24 h in the case of 2nd treatment as compared to 1st treatment after comparison. However, the tolerant line, PI254744 (control treatment) manifested higher expression values at 3d and 6d for all *BSK* genes (Fig. 5b). The genes *ClaBRI1, ClaBRI2, ClaBSK2, ClaBSK4, ClaBSK5, ClaBSK6* and *ClaBSK8* were expressed higher at 24 h in 2nd treatment as compared to 1st treatment. For control treatment, all *BSK* genes were expressed remarkably high at different time points.

**Fig. 6:**
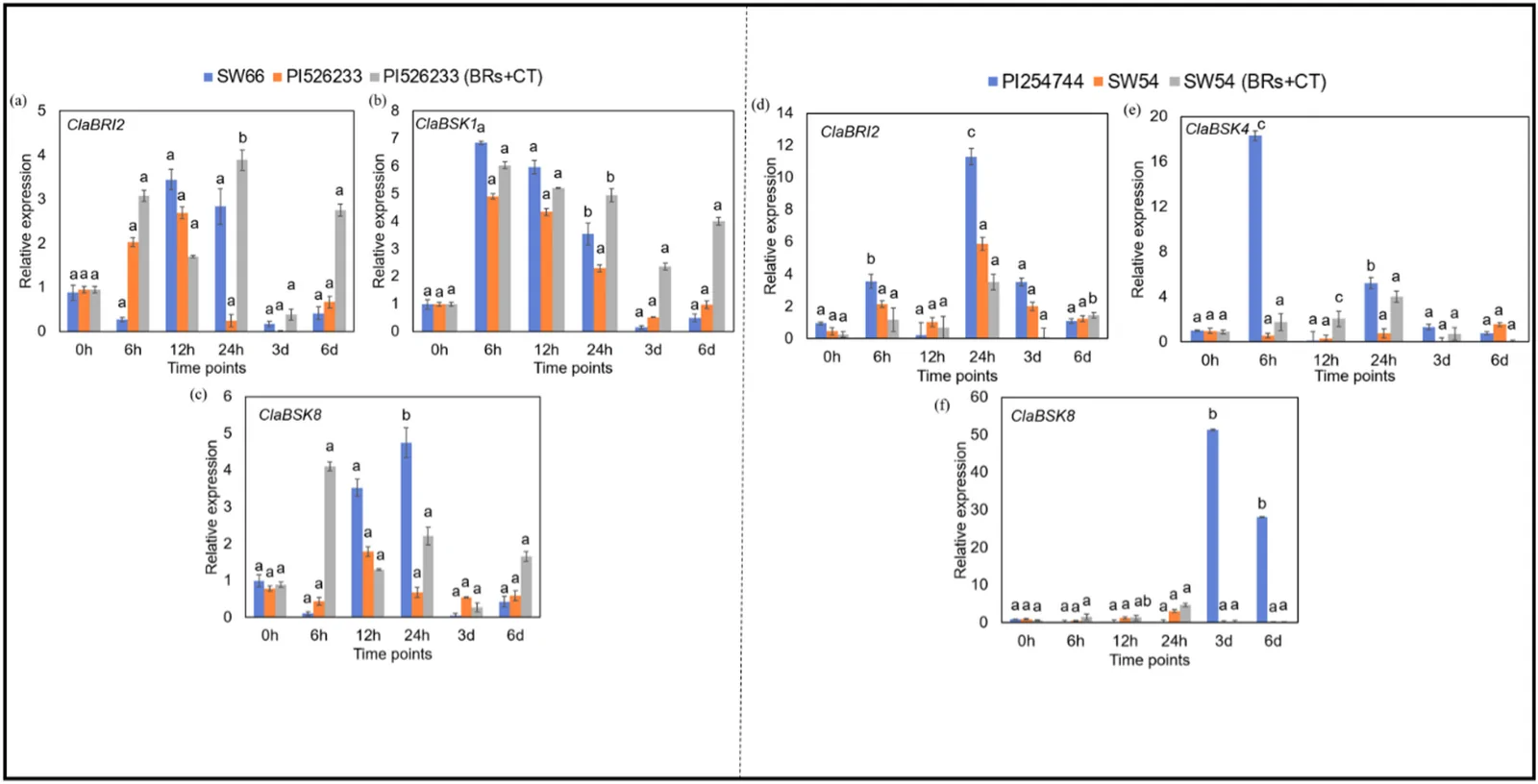
Expression patterns of *ClaBRI2* (**a**), *ClaBSK1* (**b**), *ClaBSK8* (**c**) *ClaBRI2* (**d**), *ClaBSK4* (**e**) and *ClaBSK8* (**f**) in group 1 (SW66 and PI526233) and group 2 (PI254744 and SW54) under CT and BRs + CT treatments. Blue color shows the expression of SW66 and PI254744 under CT while orange color shows the expression of PI526233 and SW54 under CT. Contrary to that, gray color shows the expression of PI526233 and SW54 under BRs + CT. The RNA samples were collected at 0h, 6h, 12h, 24h, 3d and 6d after the corresponding treatment, respectively. The error bars indicate the standard deviations of the three independent RT-qPCR biological replicates at every time point (X: time points; Y: relative expression). Different lowercase letters in the figures indicate significant differences among treatments within each sampling time point (*p* = 0.05)

It may explain why the PI254744 and SW66 were the tolerant, because of their sudden reaction to cold treatment in earlier times as compared to sensitive lines, SW54 and PI526233 which replied lately and showed some resistance under CT + BRs. Accordingly, the higher expressions of these genes were found in 2nd treatments (BRs + CT) of both groups. Hence, the results manifest positive involvement of BRs in triggering the resistance in watermelon sensitive lines when BRs and CT treatments are simultaneous applied as compared to alone CT treatment. The most highly expressed genes in 1st, 2nd and control treatments of both groups were mentioned graphically in the Fig. 6. The genes *ClaBRI2*, *ClaBSK1* and *ClaBSK8* were tremendously expressed at 6 h, 24 h, 3d and 6d respectively, in the case of control treatment. Likewise, their expressions also positively triggered in the case of 2nd treatment (CT + BRs) in 1st group (PI526233 and SW66) (Fig. 6a–c). Contrary to that, the genes *ClaBRI2*, *ClaBSK4* and *ClaBSK8* were highly expressed at 6 h, 12 h and 24 h respectively, in the case of control treatment whereas the positive response of these genes was also triggered successfully at 6 h, 12 h, 24 h and 6d respectively in the case of 2nd treatment after CT + BRs treatment in 2nd group (PI254744 and SW54) (Fig. 6d–f).

**Fig. 7:**
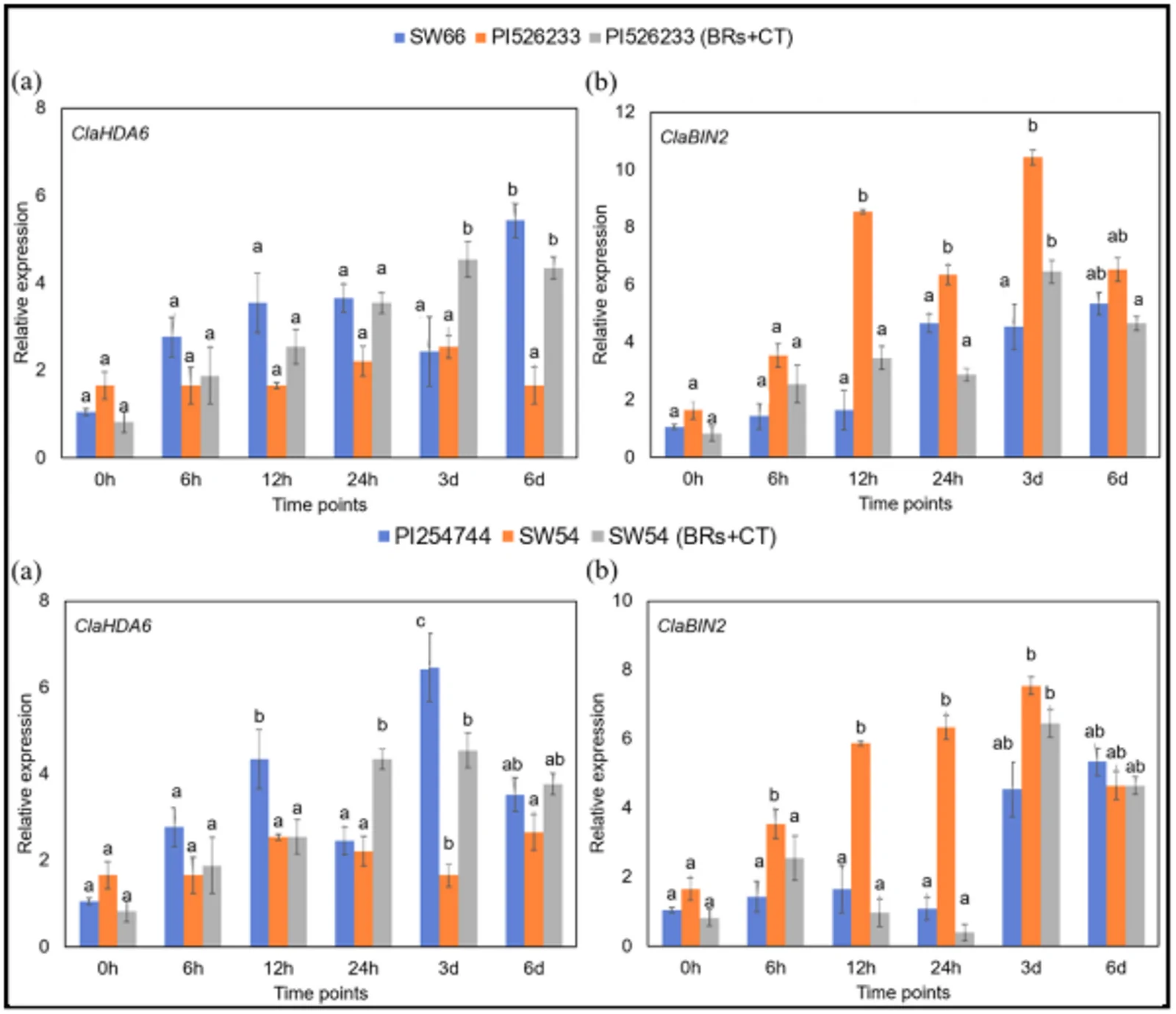
Expression patterns of *ClaHDA6* (**a**) and *ClaBIN2* (**b**) genes in group 1 (SW66 and PI526233) and group 2 (PI254744 and SW54) under CT and BRs + CT treatments. Blue color shows the expressions of SW66 and PI254744 under CT while orange color shows the expression of PI526233 and SW54 under CT. Contrary to that, gray color shows the expression of PI526233 under BRs + CT. The RNA samples were collected at 0h, 6h, 12h, 24h, 3d and 6d after the corresponding treatment, respectively. The error bars indicate the standard deviations of the three independent RT-qPCR biological replicates at every time point (X: time points; Y: relative expression). Different lowercase letters in the figures indicate significant differences among treatments within each sampling time point (*p* = 0.05)

In addition, *ClaHDA6* was found to be higher in all time points for both groups tolerant and sensitive lines under CT and BRs + CT. However, its expression was lowered at all-time points in the case of sensitive lines under CT for both groups. Contrary to that, *ClaBIN2* was expressed at increased amount at all-time points in the case of sensitive lines for both groups under CT while its expression was found higher at later times in tolerant and sensitive lines under CT and BRs + CT due to the attenuation of *CBF* genes after imitating the cold tolerance. However, its expression became lower during 0–24 h for tolerant and sensitive lines under CT and BRs + CT because of its negative response to the cold stress (Fig. 7).

### POD analysis

**Fig. 8:**
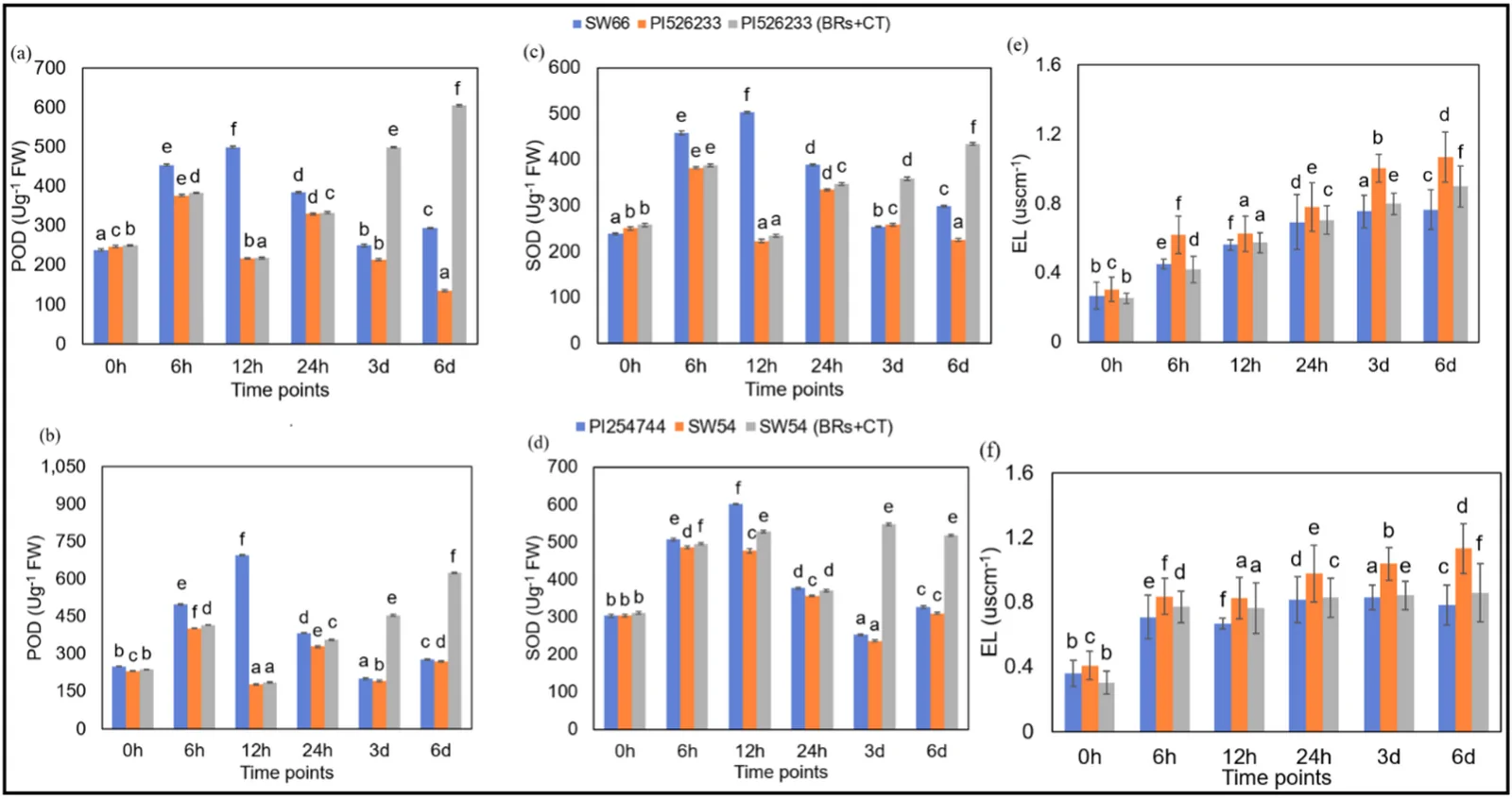
The POD, SOD and electrolyte leakage level was measured in both groups, group 1 (SW66 and PI526233) (**a**, **c**, **e**) and group 2 (PI254744 and SW54) (**b**, **d**, **f**) under CT and BRs + CT treatments. Blue color shows the expressions of SW66 and PI254744 under CT while orange color shows the expression of PI526233 and SW54 under CT. Contrary to that, gray color shows the expression of PI526233 and SW54 under BRs + CT. The RNA samples were collected at 0 h, 6 h, 12 h, 24 h, 3d and 6d after the corresponding treatment, respectively. The error bars indicate the standard deviations of the three independent biological replicates at every time point. Different lowercase letters in the figures indicate significant differences among treatments within each sampling time point (*p* = 0.05)

The POD activity was altered with the same manner in both groups, initially increased rapidly then decreased expeditiously for control treatments while the activity was positively enhanced to peak level for 2nd treatment (Fig. 8a, b). However, the POD activity was altered in zigzag manner and it was decreased at later periods in 1st treatment. For both groups, the POD activity at 12 h showed strongest level for tolerant lines especially in 2nd group tolerant line (PI254744), raising by 695 Ug^−1^ FW and 497 Ug^−1^ FW, respectively, compared to other treatments. However, with the extension of treatment time, it dropped back to the initial level, 134 Ug^−1^ FW and 276.4 Ug^−1^ FW at 6d. At 6 h of 2nd treatment, it was increased initially at the rate of 382.2 Ug^−1^ FW and 414.7 Ug^−1^ FW, suddenly decreased again to 216.3 Ug^−1^ FW and 184.3 Ug^−1^ FW at 12 h while it was gradually enhanced to the peak level at 6d with level of 605.3 Ug^−1^ FW and 622 Ug^−1^ FW as compared to the other treatments. In addition, the POD level at 6 h was 375.8 Ug^−1^ FW and 401.1 Ug^−1^ FW, then dropped back to 215.8 Ug^−1^ FW and 176 Ug^−1^ FW at 12 h, again increased to 328 Ug^−1^ FW and 327 Ug^−1^ FW at 24 h and gradually decreased at later periods for 1st treatment. In short, the POD activity was increased in both groups cold tolerant lines at CT while it showed lower trend in the case of both groups sensitive lines at CT. However, the both groups sensitive lines at BRs + CT showed increasing trend of POD activity. The enhanced POD activity under BR treatment contributes to the cellular antioxidant capacity, similar to stress-induced protective proteins (Wang et al. 2023a, b).

### SOD analysis

SOD activity varied with the same fashion in both groups, initial elevated fast then fell expeditiously for control treatments while the activity was positively boosted to peak level for 2nd treatment (Fig. 8c, d). However, the SOD activity was altered in zigzag way and it was lowered at later periods in 1st treatment. For both groups, the SOD activity at 12 h showed strongest level for tolerant lines especially in 2nd group tolerant line (PI254744), raising by 601 Ug^−1^ FW and 502 Ug^−1^ FW, respectively, compared to other treatments. However, with the extension of treatment time, it dropped back to the initial level, 327 Ug^−1^ FW and 298 Ug^−1^ FW at 6d. At 6 h of 2nd treatment, it was increased initially at the rate of 495 Ug^−1^ FW and 387 Ug^−1^ FW, suddenly decreased again at 12 h while it was gradually enhanced to the peak level at 6d with level of 518 Ug^−1^ FW and 434 Ug^−1^ FW as compared to the other treatments. In addition, the SOD level at 6 h was 485 Ug^−1^ FW and 382 Ug^−1^ FW, then dropped back to 476 Ug^−1^ FW and 223 Ug^−1^ FW at 12 h and gradually decreased at later periods for 1st treatment. In summary, both groups’ cold-tolerant lines had higher SOD activity at CT, whereas both groups’ sensitive lines had a lower trend. However, the both groups sensitive lines at BRs + CT revealed increasing trend of SOD activity. The coordinated upregulation of SOD and POD under BR treatment aligns with broader stress response mechanisms where antioxidant enzymes mitigate oxidative damage (Lou et al. 2024).

### Electrolyte leakage (EL) analysis

The EL sketch was observed almost same in both groups. However, in 1st group some exception present where the EL was found lower at initial periods in 2nd treatment as compared to control and 1st treatments (Fig. 8e, f). However, overall general trend (for sensitive line ˄, for tolerant line˅) was observed in both groups. However, the treatment under BRs + CT (2nd treatment) showed less EL compared to 1st treatment while it showed more EL when compared to control treatments. For both groups, the EL level was observed minimum at all-time points with the rate of 0.36 µscm^−1^ and 0.54 µscm^−1^ at 6 h to 0.53 µscm^−1^ and 0.69 µscm^−1^ at 6d in the case of control treatment. However, it was found a bit higher in the case of 2nd treatment with the rate of 0.63 µscm^1^ at 6 h and ended at 0.73 µscm^− 1^ after 6d while it was exceptionally found lower than control treatment, 0.29 µscm^−1^ at 6 h and then went to the 0.67 µscm^−1^ after 6d. This may be due to the brassinosteroid generally improves the membrane stability under cold stress in sensitive lines by reducing the ET leve. In addition, the EL amount was observed higher in the case of 1st treatment as compared to remaining treatments at the rate of 0.47 µscm^−1^ and 0.68 *µ*scm^− 1^ for 6 h to 0.68 µscm^−1^ and 0.81 µscm^1^ for 6d. In summary, both groups’ cold-tolerant lines had lowered EL level at CT, whereas both groups’ sensitive lines had a higher trend of EL. However, the both groups sensitive lines at BRs + CT revealed the lower trend of EL amount.

## Discussion

There are several ways that cold temperatures affect plants. Low temperatures during the seedling growth stage can cause a variety of intricate signs of cell malfunction, including wilting, growth restriction, yellowing, and even mortality (Wang et al. 2022). At different phases of growth and development, different tissues react differently to low temperatures. Because the shoot tip is so sensitive to low temperatures, the mass and growth rate of the leaves may suffer (Yaseen et al. 2025; Engels 1994). In other tests, however, cold stress on the shoot tip resulted in delayed leaf development but had no discernible effect on photosynthetic efficiency (Stone et al. 1999). As a result of a longer cell cycle and fewer cells in the seedlings, freezing stress inhibits the growth of leaves (Sowiński et al. 2005). In this study, it was also found that low temperature significantly reduced the growth rate (leaf and stem size) and showed curling of leaves in earlier times while wilting and the whole plant death displayed in the later stages of watermelon sensitive lines (PI526233 and SW54). However, the opposite growth rate was observed in the tolerant lines (PI254744 and SW66) under CT except some leaves showed curling in later stages. Under BRs + CT treatment to sensitive lines, the normal growth rate and healthy leaves condition were observed at all-time points like in previous crops e.g. maize, rice and wheat (Fig) (Rymen et al. 2007; Fujii and Saka 2001).

Excessive accumulation of reactive oxygen species (ROS), including superoxide, hydrogen peroxide, singlet oxygen, and hydroxyl radicals, contributes to cold-induced oxidative damage in plants (Singh et al. 2012; Mauch-Mani and Mauch 2005). Plants mitigate ROS accumulation through enzymatic antioxidants such as superoxide dismutase (SOD), peroxidase (POD), catalase (CAT), and ascorbate peroxidase (APX), as well as non-enzymatic antioxidants including ascorbate and glutathione (Wise and Naylor 1987; Ahamed et al. 2012; Gapper and Dolan 2006; Zhou et al. 2005; Wang et al. 2023a, b). Consistent with these findings, our study showed increased SOD and POD activities in cold-tolerant watermelon lines under CT and in sensitive lines treated with BRs + CT, suggesting enhanced antioxidant defense following BR application. The protective effects of exogenous BRs on antioxidant enzyme activities are consistent with other stress-enhancing compounds (Yu-Hao et al. 2025). Similar BR-mediated stimulation of antioxidant enzymes has been reported in *Chorispora bungeana*, maize, and other plants exposed to abiotic stresses (Liu et al. 2009; Xi et al. 2013; Sharma et al. 2019; Li et al. 2018). Notably, the 0.1 mg L⁻¹ BL treatment produced a pronounced increase in antioxidant enzyme activities in maize under chilling stress, which is consistent with the enhanced antioxidant activity observed at the same concentration in our study. These results suggest that an appropriate BRs concentration may strengthen antioxidant defense and contribute to improved cold tolerance. The transcript levels of ET biosynthesis genes (*CsACS1*, *CsACS2*, *CsACS3*, *CsACO1*, and *CsACO2*) and *CsAOX* increased after BL treatment (Słomnicka et al. 2025). According to a recent study, Cucumber Antioxidant Activity Divergence 1 (CAD1) also increases the antioxidant activity of cucumbers by transcriptionally activating genes involved in flavonoid synthesis (Xia et al. 2025).

Brassinosteroids (BRs) are important phytohormones involved in plant growth and development and contribute to plant adaptation to both biotic and abiotic stresses. Exogenous BR application has been reported to enhance plant tolerance to several abiotic stresses, including cold stress, by alleviating stress-induced physiological and molecular damage (Dong et al. 2019; Du et al. 2013). The canonical BR signaling pathway involves several important components, including the BR receptor BRI1, the co-receptor BAK1, BR-signaling kinases such as BSKs and BIN2, and the transcription factor BZR1. Previous studies have shown that BR signaling can interact with cold-response pathways through components such as BZR1, BIN2, CBF, and ICE1, suggesting an important connection between BR signaling and cold-stress responses (Li et al. 2017; Ye et al. 2019). BRI1 perceives BRs and initiates a phosphorylation cascade involving downstream signaling components, including BSK proteins, ultimately regulating transcriptional and physiological responses (Vert et al. 2005; Sun et al. 2021). The phosphorylation cascades mediated by BRI1 and BSKs in BR signaling (Sun et al. 2021) share mechanistic parallels with other receptor kinase systems that regulate stress responses.

Evidence from species other than watermelon further suggests that *BSK* genes may contribute to plant responses to environmental stresses. For example, expression patterns of *SoBSK* genes in *Spinacia oleracea* were altered under heat and cold stress, and most *SoBSK* genes showed higher expression in the heat-tolerant cultivar Sp75 than in the heat-sensitive cultivar Sp73 under heat stress (Li et al. 2022). Similarly, Arabidopsis *BSK1* and *BSK3* responded significantly to salt, cold, and heat stresses at different time points (Li et al. 2019). These observations indicate that BSK genes are not exclusively associated with BR-mediated growth regulation but may also participate in broader stress-response networks.

Nevertheless, experimental data on the contribution of particular *BSK* genes to cold-stress responses in watermelon is still limited, despite the proven involvement of BR signaling in stress adaptation in model plants and other crop species. To the best of our knowledge, the current study shows that *ClaBSK* genes respond differently to cold stress in watermelon, suggesting that these genes might be involved in the watermelon’s response to low temperatures. The differential expression patterns observed among the watermelon genotypes further suggest that individual *ClaBSK* genes may be associated with genotype-dependent differences in cold-stress responses. Nevertheless, additional functional research, such as gene overexpression, CRISPR/Cas-mediated knockout, or complementation analysis, will be necessary to ascertain the precise roles of individual ClaBSKs in watermelon cold tolerance because gene expression alone does not establish a direct regulatory or functional relationship.

The involvement of HDA6 in BR signaling and cold stress represents an additional regulatory layer, analogous to the multi-level control of stress responses (Zhang et al. 2022d). Histone deacetylase HDA6 has been implicated in freezing tolerance and has been reported to regulate BIN2 activity, providing a potential link between chromatin regulation and BR-mediated stress signaling (Eremina et al. 2016; Hao et al. 2016). In addition, Arabidopsis SHAGGY-like kinases (ASKs), including ASK21/BIN2, negatively regulate BR signaling by modulating the phosphorylation status and activity of BZR1 (Ye et al. 2019). The balance between kinase and phosphatase activities, as seen in the BRI1-BSK-BSU1-BIN2 cascade (Ge et al. 2026), is also critical for signal transduction under stress.

It is unclear, nevertheless, if the HDA6–BIN2–BZR1 regulatory module functions similarly in watermelon under cold stress. Moreover, only the expression pattern of this network in watermelon were found similar to the Arabidopsis studies mentioned above. The current study’s observation of differential expression of *ClaBSK* genes suggests that components running upstream in the BR signaling network may react to cold stress in watermelon. However, *ClaBSKs* and downstream genes like *HDA6*, *BIN2*, and *BZR1* do not directly interact, according to our expression data. Therefore, these pathways should be considered potential regulatory mechanisms rather than experimentally confirmed mechanisms in watermelon.

**Fig. 9:**
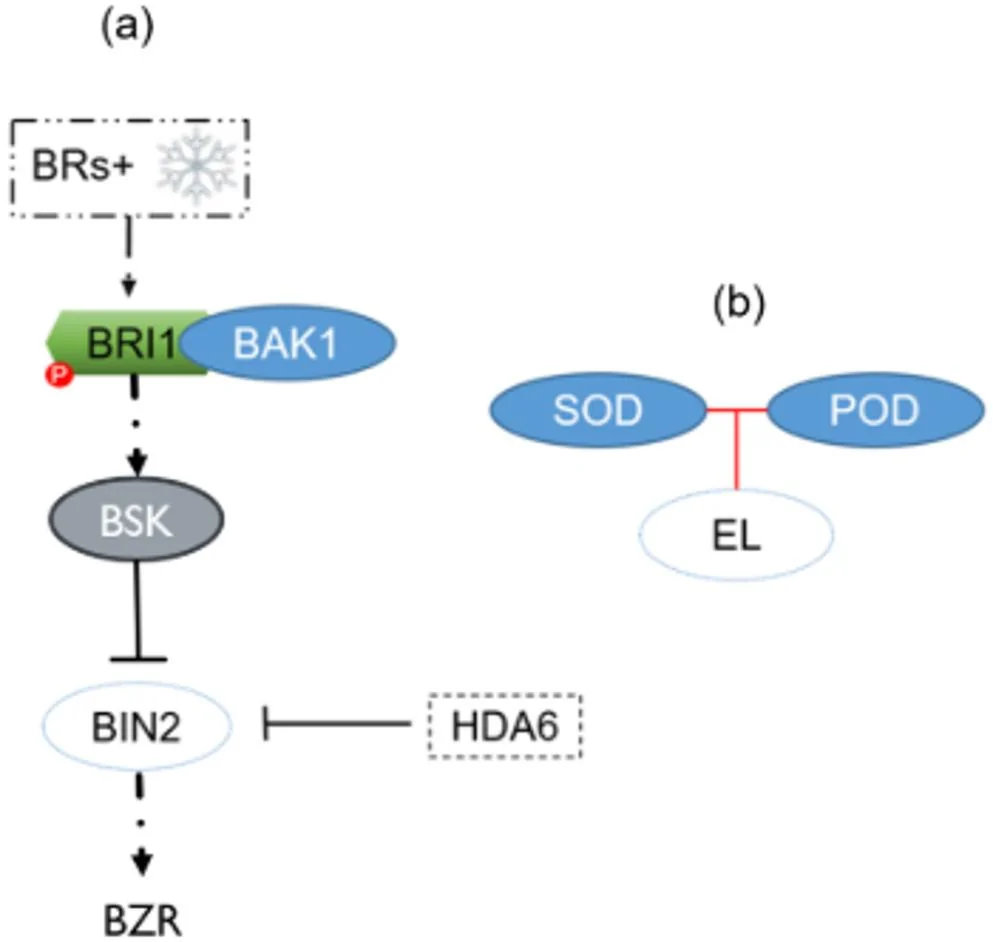
Molecular and biochemical aspects of this study. BRs regulated downstream signaling cascades, including primary PKs, particularly BRI1 and BSK which further link with BIN2 and HDA6 genes under cold treatment. However, the alternative regulation of *BIN2* and *HDA6* genes ultimately trigger the BZR TFs (**a**) and the studied biochemical components were SOD, POD and EL (**b**)

All of the PKs mentioned above, such as BRI1, BSK, and HDA6, are implicated in the CBF-dependent regulation of tolerance by BZR TFs during cold stress in the form of phosphorylation patterns (Fig. 9). However, this research also proved like a recent study that to reduce the abundance of dephosphorylated BZR1, which was already sufficient to phosphorylate with CBF genes, the BIN2 gene was downregulated in earlier phases and subsequently upregulated in later intervals (Ye et al. 2019). The regulation of transcription factor activity through phosphorylation, as demonstrated for CREB (Zhang et al. 2025), is analogous to the control of BZR1 by BIN2 in BR signaling. Furthermore, in the case of both group tolerant lines, higher expressions of these TFs were seen earlier, whereas in the case of both groups’ sensitive lines under BR + CT, higher expressions were seen later. This is because BR treatment may not simply restore the membrane stability of susceptible genotypes to the level of tolerant genotypes; rather, exogenous BR application may further enhance membrane stability under CT stress by reducing membrane damage and electrolyte leakage. Recent studies discover that plants integrate conserved signaling pathways with combination-specific regulatory modules (CSRMs) across multiple regulatory layers to mediate stress tolerance by the assistance of specific transcription factor complexes that decode unique stress combinations. For example, MFSCs are sensed through conserved signaling hubs, including ROS, calcium (Ca^2+^), and phytohormones, which provide rapid and broad responses These signals are subsequently integrated by CSRMs, e.g. TF combinatorics such as bHLH-NAC or WRKY (a negative regulator) conferring specificity to distinct stress combinations (Raza et al. 2026a, b). In previous study, it was found that during cold stress, the EL steadily increased in postharvest tomato fruits and bamboo shoots. On the other hand, BR pretreatment successfully decreased the amount of EL after 12 h (Aghdam et al. 2012; Li et al. 2012; Liu et al. 2016). Similarly, this research also showed the same outcomes for EL level in the case of both tolerant and sensitive lines under CT and BRs + CT.

## Conclusion

In conclusion, cold stress significantly inhibited seedling growth in the cold-sensitive watermelon lines PI526233 and SW54, whereas the tolerant lines PI254744 and SW66 maintained relatively normal growth under the tested cold-stress conditions. Exogenous application of BL at 0.1 mg L⁻¹ alleviated cold-induced damage in the sensitive lines, as indicated by increased SOD and POD activities and reduced electrolyte leakage compared with the corresponding cold-treated plants without BR application. The tolerant lines also exhibited higher antioxidant enzyme activities under cold stress, supporting the association between enhanced antioxidant defense and cold tolerance. Expression analysis further revealed differential and time-dependent responses of *ClaBRI-ClaBSK* related genes under cold stress and BR treatment. In addition, the contrasting expression patterns of *ClaHDA6* and *ClaBIN2* suggest that these components may be associated with BR-related signaling during the cold response. Overall, our results reveal preliminary molecular and physiological evidence that exogenous BL might partially mitigate cold-induced damage in sensitive genotypes under the experimental conditions and that BR-associated signaling may contribute to cold-stress responses in watermelon. However, further functional validation is needed to determine the precise functions of the *ClaBSK*, *ClaBRI*, *ClaHDA6*, and *ClaBIN2* genes as well as their possible use as breeding targets. Similarly, even though 0.1 mg L⁻¹ BL demonstrated positive effects in this study, more research is needed to determine its practical agricultural application under greenhouse and field conditions, while considering the factors like application method, dosage, treatment frequency, cost-effectiveness, and potential effects on growth, yield, and fruit quality.

## Supplementary Information

Below is the link to the electronic supplementary material.

Supplementary Material 1

Supplementary Material 2

Supplementary Material 3

## Data Availability

The authors declare that the data supporting the findings of this study are available within the paper.

## References

[CR1] Adhikari L, Baral R, Paudel D, Min D, Makaju SO, Poudel HP, Cold (2022) stress in plants: strategies to improve cold tolerance in forage species. Plant Stress 4:100081. 10.1016/j.stress.2022.100081

[CR2] Aghdam MS, Asghari M, Farmani B, Mohayeji M, Moradbeygi H (2012) Impact of postharvest brassinosteroids treatment on PAL activity in tomato fruit in response to chilling stress. Sci Hortic 144:116–120

[CR3] Ahamed GJ, Yuan HL, Ogweno JO, Zhou Y, Xia X, Mao WH, Shi K, Yu J (2012) Brassinosteroid alleviates phenanthrene and pyrene phytotoxicity by increasing detoxification activity and photosynthesis in tomato. Chemosphere 86:546–555 10.1016/j.chemosphere.2011.10.038 22119279

[CR4] Assefa AD, Hur O, Ro N, Lee J, Hwang A, Kim B (2020) Fruit morphology, citrulline, and arginine levels in diverse watermelon (*Citrullus lanatus*) germplasm collections. Plants 9:1054. 10.3390/plants9091054 PMC7569901 32824928

[CR5] Chen C, Cheng D, Li L, Sun X, He S, Li M, Chen J (2023) Physiological characteristics and transcriptome analysis of exogenous brassinosteroid-treated Kiwifruit. Int J Mol Sci 24:19 10.3390/ijms242417252 PMC10744020 38139080

[CR6] Chen Y, Ye T, Tang S, Hu P (2025) Facing chilling, kinase-transcription factors relay cold tolerance signals. New Crops 2:100058

[CR7] Clouse SD (2011) Brassinosteroid signal transduction: from receptor kinase activation to transcriptional networks regulating plant development. Plant Cell 23:1219–1230 10.1105/tpc.111.084475 PMC3101532 21505068

[CR8] Ding Y, Lv J, Shi Y, Gao J, Hua J, Song C (2019) Egr2 phosphatase regulates ost1 kinase activity and freezing tolerance in Arabidopsis. Embo J 38:e99819. 10.15252/embj.201899819 PMC6315290 30429206

[CR9] Dong SK, Jiang YZ, Dong YC, Wang LB, Wang WJ, Ma ZZ, Yan C, Ma CM, Liu LJ 2019 A study on soybean responses to drought stress and rehydration. Saudi J Biol Sci 26 (8):2006–2017 10.1016/j.sjbs.2019.08.005 PMC6923469 31889786

[CR10] Du H, Liu HB, Xiong LZ (2013) Endogenous auxin and jasmonic acid levels are differentially modulated by abiotic stresses in rice. Front Plant Sci 4:397 10.3389/fpls.2013.00397 PMC3793129 24130566

[CR11] Engels C (1994) Effect of root and shoot meristem temperature on shoot to root dry matter partitioning and the internal concentrations of nitrogen and carbohydrates in maize and wheat. Ann Bot 73:211–219

[CR12] Eremina M, Unterholzner SJ, Rathnayake AI, Castellanos M, Khan M, Kugler KG, May ST, Mayer KFX, Rozhon W (2016a) Brassinosteroids participate in the control of basal and acquired freezing tolerance of plants. Proc Natl Acad Sci USA 113:E5982–E5991 10.1073/pnas.1611477113 PMC5056081 27655893

[CR13] Fujii S, Saka H (2001) The promotive effect of brassinolide on lamina joint-cell elongation, germination and seedling growth under low-temperature stress in rice (*Oryza sativa* L). Plant Prod Sci 4:210–214

[CR14] Gapper C, Dolan L (2006) Control of plant development by reactive oxygen species1. Plant Physiol 141:341–345 10.1104/pp.106.079079 PMC1475470 16760485

[CR15] Ge C, Shi Z, He J, Feng X, Shang K, Liao X, Liu S (2026) Cyanoglycosides isolated from Moringa oleifera seeds inhibited PFKFB3/TGF-β1/smads pathway to alleviate diabetic nephropathy through driving metabolic reprogramming. Pharm Biol 64(1):130–142 10.1080/13880209.2025.2607563 PMC12777776 41466459

[CR16] Guan YL, Hwarari D, Mateko KH, Baseer A, Cao YW, Movahedi A (2023) Low temperature stress-induced perception and molecular signaling pathways in plants. Environ Exp Bot 207:105190. 10.1016/J.ENVEXPBOT.2022.105190

[CR17] Gusain S, Joshi S, Joshi R (2023) Sensing, signaling, and regulatory mechanism of cold-stress tolerance in plants. Plant Physiol Biochem 197:107646. 10.1016/j.plaphy.2023.107646 36958153

[CR18] Hao Y, Wang H, Qiao S, Leng L, Wang X (2016) Histone deacetylase HDA6 enhances brassinosteroid signaling by inhibiting the BIN2 kinase. Proc Natl Acad Sci USA 113:10418–10423 10.1073/pnas.1521363113 PMC5027435 27562168

[CR19] Jiang B, Shi Y, Peng Y, Jia Y, Yan Y (2020) Dong X Cold-induced cbfpif3 interaction enhances freezing tolerance by stabilizing the phyb thermosensor in Arabidopsis. Mo Plant 13:894–906. 10.1016/j.molp.2020.04.006 32311530

[CR20] Jin C, Li KQ, Xu XY, Zhang HP, Chen HX, Chen YH (2017) A novel nac transcription factor, pbenac1, of *pyrus betulifolia* confers cold and drought tolerance via interacting with pbedrebs and activating the expression of stress responsive genes. Fron. Plant Sc 8:1049. 10.3389/fpls.2017.01049 PMC5491619 28713394

[CR21] Kamble NU (2024) Histone deacetylase oshda716 chilling out with osbzip46: antagonistically regulating cold stress tolerance in rice. Plant Cell 36:1592–1593. 10.1093/plcell/koae025 PMC11062439 38267564

[CR22] Kidokoro S, Hayashi K, Haraguchi H, Ishikawa T, Soma F, Konoura L (2021) Posttranslational regulation of multiple clock-related transcription factors triggers cold-inducible gene expression in arabidopsis. Proc Natl Acad Sci U S A 118:e2021048118. 10.1073/pnas.2021048118 PMC7958436 33649234

[CR23] Kidokoro S, Konoura I, Soma F, Suzuki T, Miyakawa T, Tanokura M (2023) Clock-regulated coactivators selectively control gene expression in response to different temperature stress conditions in arabidopsis. Proc Natl Acad Sci U S A 120:e2078784176. 10.1073/pnas.2216183120 PMC10120023 37036986

[CR26] Kim TW, Wang ZY (2010) Brassinosteroid signal transduction from receptor kinases to transcription factors. In: Merchant S, Briggs WR, Ort D (eds) Annual Review of Plant Biology, vol 61. Palo Alto, CA, USA, Annual Reviews 10.1146/annurev.arplant.043008.092057 20192752

[CR25] Kim TW, Guan S, Sun Y, Deng Z, Tang W, Shang JX, Sun Y, Burlingame AL, Wang ZY (2009) Brassinosteroid signal transduction from cell-surface receptor kinases to nuclear transcription factors. Nat Cell Biol 11:1254–1260 10.1038/ncb1970 PMC2910619 19734888

[CR24] Kim TW, Guan S, Burlingame AL, Wang ZY (2011) The CDG1 kinase mediates brassinosteroid signal transduction from BRI1 receptor kinase to BSU1 phosphatase and GSK3-like kinase BIN2. Mol Cell 43:561–571 10.1016/j.molcel.2011.05.037 PMC3206214 21855796

[CR27] Lee ES, Park JH, Wi SD, Kang CH, Chi YH, Chae HB (2021) Redox-dependent structural switch and cbf activation confer freezing tolerance in plants. Nat Plants 7:914–922. 10.1038/s41477-021-00944-8 34155371

[CR28] Li B, Zhang C, Cao B, Qin G, Wang W, Tian S (2012) Brassinolide enhances cold stress tolerance of fruit by regulating plasma membrane proteins and lipids. Amino Acids 43(6):2469–2480 10.1007/s00726-012-1327-6 22660900

[CR29] Li H, Ye KY, Shi YT, Cheng JK, Zhang XY, Yang SH (2017) BZR1 positively regulates freezing tolerance via CBF-dependent and CBF-independent pathways in Arabidopsis. Mol Plant 10(4):545–559 10.1016/j.molp.2017.01.004 28089951

[CR30] Li X, Wei JP, Ahammed GJ, Zhang L, Li Y, Yan P, Zhang LP, Han WY (2018) Brassinosteroids attenuate moderate high temperature-caused decline in tea quality by enhancing theanine biosynthesis in *Camellia sinensis* L. front. Plant Sci 9:1016 10.3389/fpls.2018.01016 PMC6066615 30087682

[CR32] Li Z, Shen J, Liang J (2019) Genome-wide identification, expression profile, and alternative splicing analysis of the brassinosteroid-signaling kinase (BSK) family genes in Arabidopsis. Int J Mol Sci 20(5):1138 10.3390/ijms20051138 PMC6429265 30845672

[CR38] Li J, Wang T, Han J, Ren Z (2020) Genome-wide identification and characterization of cucumber bHLH family genes and the functional characterization of CsbHLH041 in NaCl and ABA tolerance in Arabidopsis and cucumber. BMC Plant Biol 20(1):272 10.1186/s12870-020-02440-1 PMC7291561 32527214

[CR31] Li Y, Zhang H, Zhang Y, Liu Y, Li Y, Tian H, Dai S (2022) Genome-wide identification and expression analysis reveals spinach brassinosteroid-signaling kinase (BSK) gene family functions in temperature stress response. BMC Genomics 23(1):453 10.1186/s12864-022-08684-5 PMC9208177 35725364

[CR33] Liu Y, Zhao Z, Si J, Di C, Han J, An L (2009) Brassinosteroids alleviate chilling-induced oxidative damage by enhancing antioxidant defense system in suspension cultured cells of *Chorispora bungeana*. Plant Growth Regul 59:207–214

[CR34] Liu Z, Li L, Luo Z, Zeng F, Jiang L, Tang K (2016) Effect of brassinolide on energy status and proline metabolism in postharvest bamboo shoot during chilling stress. Postharvest Biol Technol 111:240–246

[CR35] Liu T, Luo T, Guo X, Zou X, Zhou D, Afrin S, Luo Z (2019) PgMYB2, a MeJA-responsive transcription factor, positively regulates the dammarenediol synthase gene expression in Panax ginseng. Int J Mol Sci 20(9):2219 10.3390/ijms20092219 PMC6539309 31064108

[CR36] Liu F, Xi M, Liu T, Wu X, Ju L, Wang D (2024) The central role of transcription factors in bridging biotic and abiotic stress responses for plants’ resilience. New crops 1:100005

[CR37] Livak KJ, Schmittgen TD (2001) Analysis of relative gene expression data using real-time quantitative PCR and the 2^–∆∆CT^ method. Methods 25:402–408 10.1006/meth.2001.1262 11846609

[CR39] Lou Y, Zou X, Pan Z, Huang Z, Zheng S, Zheng X, Zhang Y (2024) The mechanism of action of Botrychium (Thunb.) Sw. for prevention of idiopathic pulmonary fibrosis based on 1H-NMR-based metabolomics. J Phar Pharmacol 76(8):1018–1027 10.1093/jpp/rgae058 38776436

[CR40] Ma Y, Dai X, Xu Y, Luo W, Zheng X, Zeng D (2015) Cold1 confers chilling tolerance in rice. Cell 160:1209–1221. 10.1016/j.cell.2015.01.046 25728666

[CR41] Mauch-Mani B, Mauch F (2005) The role of abscisic acid in plant–pathogen interactions. Curr Opin Plant Boil 8:409–414 10.1016/j.pbi.2005.05.015 15939661

[CR42] Mu D, Feng N, Zheng D, Zhou H, Liu L, Chen G (2022) Studies on the physiological mechanism of brassinolide to improve the resistance of rice seedlings to NaCl stress. Water Air Soil Pollut 233:238

[CR43] Nishiyama I (1970) Male sterility caused by cooling treatment at the meiotic stage in rice plants. IV: respiratory activity of anthers following cooling treatment at the meiotic stage. Jpn J Crop Sci 39:65–70

[CR44] Noh J, Kim JM, Sheikh S, Lee SG, Lim JH, Seong MH (2013) Effect of heat treatment around the fruit set region on growth and yield of watermelon [Citrullus lanatus (Thunb.) Matsum. and Nakai]. Physiol Mol Biol Plants 19:509–514. 10.1007/s12298-013-0174-6 PMC3781286 24431519

[CR45] Raza A, Bhardwaj S, Rahman MA, García-Caparrós P, Habib M, Saeed F, Varshney RK (2024) Trehalose: a sugar molecule involved in temperature stress management in plants. Crop J 12(1):1–16

[CR46] Raza A, Li Y, Charagh S, Guo C, Zhao M, Hu Z (2026a) Melatonin-enabled omics: understanding plant responses to single and combined abiotic stresses for climate-smart agriculture. GM crops food 17(1):2614130 10.1080/21645698.2026.2614130 PMC12851399 41589633

[CR47] Raza A, Li Y, Zhao M, Ming T, Ortiz R, Henry RJ, Hu Z (2026b) Distinct regulatory networks mediate plant adaptation to multifactorial abiotic stress combinations: rethinking of plant stress genetics. Plant Stress 101458

[CR48] Rodríguez VM, Soengas P, Alonso-Villaverde V, Sotelo T, Cartea ME, Velasco P (2015) Effect of temperature stress on the early vegetative development of *Brassica oleracea* L. BMC Plant Biol 15:145. 10.1186/s12870-015-0535-0 PMC4467057 26077340

[CR49] Rymen B, Fiorani F, Kartal F, Vandepoele K, Inzé D, Beemster GTS (2007) Cold nights impair leaf growth and cell cycle progression in maize through transcriptional changes of cell cycle genes1[W][OA]. Plant Physiol 143:1429–1438 10.1104/pp.106.093948 PMC1820914 17208957

[CR50] Sharma A, Shahzad B, Kumar V, Kohli SK, Sidhu GPS, Bali AS, Handa N, Kapoor D, Bhardwaj R, Zheng B (2019) Phytohormones regulate accumulation of osmolytes under abiotic stress. Biomolecules 9:285 10.3390/biom9070285 PMC6680914 31319576

[CR51] Shirani Bidabadi S, Mehralian M (2020) Arbuscular mycorrhizal fungi inoculation to enhance chilling stress tolerance of watermelon. Gesunde Pflanzen 72:171–179. 10.1007/s10343-020-00499-2

[CR52] Silverwood SM, Lichter KE, Kleiner HS, Katsumoto TR (2025) A unified strategy for addressing climate change, improving public health, and mitigating environmental degradation. Agric Commun 3:100090

[CR53] Singh I, Kumar U, Singh SK, Gupta C, Singh M, Kushwaha SR (2012) P hysiological and biochemical effect of 24-epibrassinoslide on cold tolerance in maize seedlings. Physiol Mol Boil 18:229–236 10.1007/s12298-012-0122-x PMC3550514 23814437

[CR54] Słomnicka R, Cieplak M, Martín-Hernández AM, Bartoszewski G (2025) Brassinosteroids in cucurbits: modulators of plant growth architecture and stress response. Int J Mol Sci 26(15):7234 10.3390/ijms26157234 PMC12346612 40806367

[CR55] Sowiński P, Rudzi ´nska-Langwald A, Adamczyk J, Kubica I, Fronk J (2005) Recovery of maize seedling growth, development and photosynthetic efficiency after initial growth at low temperature. J Plant Physiol 162:67–80 10.1016/j.jplph.2004.03.006 15700422

[CR56] Sreeramulu S, Mostizk Y, Sunitha S, Shani E, Nahum H, Salomon D, Hayun LB, Gruetter C, Rauh D, Ori N (2013) BSKs are partially redundant positive regulators of brassinosteroid signaling in Arabidopsis. Plant J 74:905–919 10.1111/tpj.12175 23496207

[CR57] Stone P, Sorensen I, Jamieson P (1999) Effect of soil temperature on phenology, canopy development, biomass and yield of maize in a cool-temperate climate. Field Crop Res 63:169–178

[CR58] Sun TL, Li WQ, Tong XL, Liu XY, Zhou WH (2021) Xanthohumol attenuates isoprenaline-induced cardiac hypertrophy and fibrosis through regulating PTEN/AKT/mTOR pathway. Eur J Pharmacol 891:173690 10.1016/j.ejphar.2020.173690 33127362

[CR59] Tang W, Kim TW, Oses-Prieto JA, Sun Y, Deng Z, Zhu S, Wang R, Burlingame AL, Wang ZY (2008) BSKs mediate signal transduction from the receptor kinase BRI1 in Arabidopsis. Science 321:557–560 10.1126/science.1156973 PMC2730546 18653891

[CR61] Verma V, Ravindran P, Kumar PP (2016) Plant hormone-mediated regulation of stress responses. BMC Plant Biol 16(1):86 10.1186/s12870-016-0771-y PMC4831116 27079791

[CR60] Vert G, Nemhauser JL, Geldner N, Hong FX, Chory J (2005) Molecular mechanisms of steroid hormone signaling in plants. Annu Rev Cell Dev Biol 21:177–201 10.1146/annurev.cellbio.21.090704.151241 16212492

[CR66] Wang ZY (2012) Brassinosteroids modulate plant immunity at multiple levels. Proc Natl Acad Sci USA 109:7–8 10.1073/pnas.1118600109 PMC3252925 22198764

[CR67] Wang ZY, Nakano T, Gendron J, He J, Chen M, Vafeados D, Yang Y, Fujioka S, Yoshida S, Asami T (2002) Nuclear localized BZR1 mediates brassinosteroid-induced growth and feedback suppression of brassinosteroid biosynthesis. Dev Cell 2:505–513 10.1016/s1534-5807(02)00153-3 11970900

[CR62] Wang H, Liang X, Huang J, Zhang D, Lu H, Liu Z, Bi Y (2010a) Involvement of ethylene and hydrogen peroxide in induction of alternative respiratory pathway in salt-treated Arabidopsis calluses. Plant Cell Physiol 51(10):1754–1765 10.1093/pcp/pcq134 20801923

[CR64] Wang M, Jiang W, Yu H (2010b) Effects of exogenous epibrassinolide on photosynthetic characteristics in tomato (*Lycopersicon esculentum* Mill) seedlings under weak light stress. J Agric Food Chem 58:3642–3645 10.1021/jf9033893 20178315

[CR63] Wang D, Yang Z, Wu M, Wang W, Wang Y, Nie S (2022) Enhanced brassinosteroid signaling via the overexpression of SlBRI1 positively regulates the chilling stress tolerance of tomato. Plant Sci 320:111281 10.1016/j.plantsci.2022.111281 35643607

[CR65] Wang Y, Jiang Q, Wang X, Xi Z (2023a) Brassinosteroid stimulates hydrogen peroxide biosynthesis and reduces the effect of cold stress. J Plant Growth Regul 42(6):3757–3769

[CR68] Wang YX, Yu TF, Wang CX, Wei JT, Zhang SX, Liu YW, Xu ZS (2023b) Heat shock protein TaHSP17. 4, a TaHOP interactor in wheat, improves plant stress tolerance. Int J Biol Macromol 246:125694 10.1016/j.ijbiomac.2023.125694 37414309

[CR69] Wang F, Chen S, Zhou J, Zhu R, Chen J, Wang Y (2025) ARTP mutagenesis for genome-wide identification of genes important for biofilm regulation in spoilage bacterium Pseudomonas fluorescens PF08. Appl Environ Microbiol 91(6):e00218–e00225 10.1128/aem.00218-25 PMC12175535 40530873

[CR70] Wang M, Du P, Xi L, Lin H, Zhang S (2026) Dynamic coordination: how ERF transcription factors coordinate plant development and adaptive stress responses. Biomolecules 16(3):466 10.3390/biom16030466 PMC13024231 41897400

[CR71] Waraich EA, Ahmad R, Halim A, Aziz T (2012) Alleviation of temperature stress by nutrient management in crop plants: a review. J Soil Sci Plant Nutr 12:221–244. 10.4067/S0718-95162012000200003

[CR72] Wehner TC, Naegele RP, Myers JR, Narinder PSD, Crosby K (2020) Curcubits 2:32. CABI, Wallingford

[CR73] Wise RR, Naylor AW (1987) Chilling-enhanced photooxidation: the peroxidative destruction of lipids during chilling injury to photosynthesis and ultrastructure. Plant Physiol 83:272–277 10.1104/pp.83.2.272 PMC1056347 16665235

[CR74] Xi Z, Wang Z, Fang Y, Hu Z, Hu Y, Deng M, Zhang ZW (2013) Effects of 24-epibrassinolide on antioxidation defense and osmoregulation systems of young grapevines (*V. vinifera* L.) under chilling stress. Plant Growth Regul 71:57–65

[CR75] Xia C, Yin S, Zhang J, Teng H, Li Y, Mao A, Wen C (2025) CAD1 enhances antioxidant activity to facilitate cold storage by regulating flavonoid synthesis in cucumber. Agric Commun 3(1):100071

[CR76] Yang T, Zhuang Z, Bian J, Ren Z, Ta W, Peng Y (2025) Mechanisms of exogenous brassinosteroids and abscisic acid in regulating maize cold stress tolerance. Int J Mol Sci 26(7):3326 10.3390/ijms26073326 PMC11989956 40244224

[CR77] Yaseen I, Choi S, Mukhtar T, Park JI, Kim HT (2025) Quantification of growth and physiological characteristics in tolerant and sensitive watermelon lines under cold treatment. Hort Environ Biotechnol 66(2):189–204

[CR78] Ye K, Li H, Ding Y, Shi Y, Song C, Gong Z, Yang S (2019) BRASSINOSTEROID-INSENSITIVE2 negatively regulates the stability of transcription factor ICE1 in response to cold stress in Arabidopsis. Plant Cell 31(11):2682–2696 10.1105/tpc.19.00058 PMC6881119 31409630

[CR80] Yin Y, Wang ZY, Mora-Garcia S, LiJ, Yoshida S, Asami T, Chory J (2002) BES1 accumulates in the nucleus in response to brassinosteroids to regulate gene expression and promote stem elongation. Cell 109:181–191 10.1016/s0092-8674(02)00721-3 12007405

[CR81] Yoshida T, Fàbregas N, Fernie AR (2025) Unveiling links between phytohormone and abiotic stress signaling. Plant Horm 1(1)

[CR82] Yu-Hao D, Chun C, Ping-Ping W, Chun W, Xiong F (2025) Sugarcane extract enhances stress resilience and longevity in Caenorhabditis elegans: NMR structural elucidation and transcriptomic mechanisms. Food Res Int 117863 10.1016/j.foodres.2025.117863 41360552

[CR83] Zhang B, Li S (2022b) Shui W Post-translational modifications of g protein coupled receptors revealed by proteomics and structural biology. Front Chem 10:843502. 10.3389/fchem.2022.843502 PMC8960047 35355784

[CR85] Zhang M, Zhao R, Huang K, Huang S, Wang H, Wei Z (2022a) The oswrky63-oswrky76-osdreb1b module regulates chilling tolerance in rice. Plant J 112:383–398. 10.1111/tpj.15950 35996876

[CR84] Zhang H, Zhao D, Tang Z, Zhang Y, Zhang K, Dong J, Wang F (2022c) Exogenous brassinosteroids promotes root growth, enhances stress tolerance, and increases yield in maize. Plant Signal Behav 17:2095139 10.1080/15592324.2022.2095139 PMC9255028 35775499

[CR86] Zhang L, Liu J, Cheng J, Sun Q, Zhang Y, Liu J, Cai Y (2022d) lncRNA7 and lncRNA2 modulate cell wall defense genes to regulate cotton resistance to Verticillium wilt. Plant Physiol 189(1):264–284 10.1093/plphys/kiac041 PMC9070856 35134243

[CR87] Zhang L, Wang JM, Wang L, Zheng S, Bai Y, Fu JL, Li DWC (2025) The transcription factor CREB regulates epithelial-mesenchymal transition of lens epithelial cells by phosphorylation-dependent and phosphorylation-independent mechanisms. J Biol Chem 301(1) 10.1016/j.jbc.2024.108064 PMC11773003 39662835

[CR88] Zhao C, Wang P, Si T, Hsu CC, Wang L, Zayed O (2017) Map kinase cascades regulate the cold response by modulating ice1 protein stability. Dev Cell 43:618–629. 10.1016/j.devcel.2017.09.024 PMC5716877 29056551

[CR89] Zhou B, Guo Z, Xing J, Huang B (2005) Nitric oxide is involved in abscisic acid-induced antioxidant activities in *Stylosanthes guianensis*. J E Bot 56:3223–3228 10.1093/jxb/eri319 16263901

