# Supplementary material for: Genome wide identification, characterization of upstream protein kinases involving CBF dependent pathway and antioxidant enzyme analysis in Watermelon (Citrullus lanatus) under brassinosteroid and cold treatment
Source: Physiol Mol Biol Plants. 2026 Oct 12;32(11):2531–46. doi: 10.1007/s12298-026-01824-1 (PMC13759928; doi:10.1007/s12298-026-01824-1)
Supplement: Supplementary file 1 [file 12298_2026_1824_MOESM1_ESM.docx]

| **SL.** | **Gene name** | **Gene ID** | **Primer** | **Sequences (5’ ^…^ 3’)** | **Annealing temperature** |
| --- | --- | --- | --- | --- | --- |
| 1 | ClaBRI2 | Cla97C01G014900 | Forward primer | ACCCTCTTTCCCATCTTCTTC | 59-60 ^o^C |
|  |  |  | Reverse Primer | GAAGGGTTGGGTTTGGAAGT |  |
| 2 | ClaBRI1 | Cla97C01G007180 | Forward primer | CTTCCTCTTGCTTTGCCAGT |  |
|  |  |  | Reverse Primer | TCCAACTGGACAACACTCCA |  |
| 3 | ClaBSK2 | Cla97C02G036790.1 | Forward primer | GAAAAGGTTCAAACCCAGCA |  |
|  |  |  | Reverse Primer | ATTGTCGGTAGCAGCCTTCA |  |
| 4 | ClaBSK8 | Cla97C08G152340.1 | Forward primer | GGTTGCTGTGGATCGTCTTT |  |
|  |  |  | Reverse Primer | AGGAGAACTCGGAGAAAGCA |  |
| 5 | ClaBSK6 | Cla97C05G096280.1 | Forward primer | GCTGTTGCCAATCCTCGTTA |  |
|  |  |  | Reverse Primer | AGCGAAGGAAAACTCCACAA |  |
| 6 | ClaBSK4 | Cla97C04G074030.1 | Forward primer | CTCGTTTTGCTGGTTCCATT |  |
|  |  |  | Reverse Primer | TGCTCCGACACTATGTTCTCC |  |
| 7 | ClaBSK3 | Cla97C02G045830.1 | Forward primer | GAGCTCGTTGCTCCAGATTT |  |
|  |  |  | Reverse Primer | CATTAGGCGCTTTCTCTCCA |  |
| 8 | ClaBSK5 | Cla97C05G083250.1 | Forward primer | TCATCTTCCTTCCCCTGATG |  |
|  |  |  | Reverse Primer | GCGTTTAATGGCAACCAATC |  |
| 9 | ClaBSK1 | Cla97C01G002840.1 | Forward primer | GGTTGCGAGTGTTCGAAGTT |  |
|  |  |  | Reverse Primer | TGGCCGTTCTCAATTGATCT |  |
| 10 | ClaBSK7 | Cla97C07G135190.1 | Forward primer | TCAAAACTCTCAGGGTGCTGT |  |
|  |  |  | Reverse Primer | TCTCAACAGCAAATCCAGATG |  |
| 11 | ClaHDA6 | Cla97C01G001100 | Forward primer | TGGCGGTAGTCAAACCTGAG |  |
|  |  |  | Reverse Primer | CAGGATTCACTCGCTGTGGA |  |
| 12 | ClaBIN2 | Cla97C10G193440 | Forward primer | CGTCCTCTTCCAACCCACTG |  |
|  |  |  | Reverse Primer | CGATCGGTGCAGGGAGTATC |  |

**Table S1** List of primer pairs used for amplification of primary TFs which involve in triggering CBF dependent pathway.
